# IRAK1-regulated IFN-γ signaling induces MDSC to facilitate immune evasion in FGFR1-driven hematological malignancies

**DOI:** 10.1186/s12943-021-01460-1

**Published:** 2021-12-14

**Authors:** Baohuan Cai, Yun Liu, Yating Chong, Hualei Zhang, Atsuko Matsunaga, Xuexiu Fang, Rafal Pacholczyk, Gang Zhou, John K. Cowell, Tianxiang Hu

**Affiliations:** 1 Georgia Cancer Center, Augusta University, 1410 Laney Walker Blvd, GA 30912 Augusta, USA; 2 Department of Pediatrics, Tongji Hospital, Tongji Medical College, Huazhong University of Science and Technology, Wuhan, China; 3 Department of Geriatrics, Union Hospital, Tongji Medical College, Huazhong University of Science and Technology, Wuhan, China; 4 Department of Radiation Oncology, Tianjin Medical University Cancer Institute and Hospital, National Clinical Research Center for Cancer, Key Laboratory of Cancer Prevention and Therapy, Tianjin’s Clinical Research Center for Cancer, Tianjin, China

**Keywords:** Leukemia, Lymphoma, IRAK1, Immune surveillance, IFN-γ

## Abstract

**Background:**

Stem Cell leukemia/lymphoma syndrome (SCLL) presents as a myeloproliferative disease which can progress to acute myeloid leukemia and is associated with the coincident development of B-cell and T-cell lymphomas. SCLL is driven by the constitutive activation of fibroblast growth factor receptor-1 (FGFR1) as a result of chromosome translocations with poor outcome. Mouse models have been developed which faithfully recapitulate the human disease and have been used to characterize the molecular genetic events that are associated with development and progression of the disease.

**Methods:**

CRISPR/Cas9 approaches were used to generate SCLL cells null for Interleukin receptor associated kinase 1 (IRAK1) and interferon gamma (IFNG) which were introduced into syngeneic hosts through tail vein injection. Development of the disease and changes in immune cell composition and activity were monitored using flow cytometry. Bead-based immunoassays were used to compare the cytokine and chemokine profiles of control and knock out (KO) cells. Antibody mediated, targeted depletion of T cell and MDSCs were performed to evaluate their role in antitumor immune responses.

**Results:**

In SCLL, FGFR1 activation silences miR-146b-5p through DNMT1-mediated promoter methylation, which derepresses the downstream target IRAK1. IRAK1 KO SCLL cells were xenografted into immunocompetent syngeneic mice where the typical rapid progression of disease was lost and the mice remained disease free. IRAK1 in this system has no effect on cell cycle progression or apoptosis and robust growth of the KO cells in immunodeficient mice suggested an effect on immune surveillance. Depletion of T-cells in immunocompetent mice restored leukemogenesis of the KO cells, and tumor killing assays confirmed the role of T cells in tumor clearance. Analysis of the immune cell profile in mice transplanted with the IRAK1 expressing mock control (MC) cells shows that there is an increase in levels of myeloid-derived suppressor cells (MDSCs) with a concomitant decrease in CD4+/CD8+ T-cell levels. MDSC suppression assays and depletion experiments showed that these MDSCs were responsible for suppression of the T cell mediated leukemia cell elimination. Immuno-profiling of a panel of secreted cytokines and chemokines showed that activation of IFN-γ is specifically impaired in the KO cells. In vitro and in vivo expression assays and engraftment with interferon gamma receptor-1 (IFNGR1) null mice and IFNG KO SCLL cells, showed the leukemia cells produced IFN-γ directly participating in the induction of MDSCs to establish immune evasion. Inhibition of IRAK1 using pacritinib suppresses leukemogenesis with impaired induction of MDSCs and attenuated suppression of CD4+/CD8+ T-cells.

**Conclusions:**

IRAK1 orchestrates a previously unknown FGFR1-directed immune escape mechanism in SCLL, through induction of MDSCs via regulation of IFN-γ signaling from leukemia cells, and targeting IRAK1 may provide a means of suppressing tumor growth in this syndrome by restoring immune surveillance.

**Supplementary Information:**

The online version contains supplementary material available at 10.1186/s12943-021-01460-1.

## Background

Rearrangements of FGFR1, as a result of chromosome translocations, are consistently associated with the development of myeloid and lymphoid malignancies associated with SCLL syndrome. Patients typically present with a myeloproliferative disease, which can progress to AML and may be accompanied by either T-cell or B-cell malignancies [1]. There have been many different rearrangements described, where the consistent feature is the juxtaposition of the FGFR1 kinase domain adjacent to a dimerization domain in the resulting chimeric protein [1]. Unlike the parental FGFR1 kinase, the chimeric proteins are no longer tethered in the membrane and activation is independent of ligand binding. Constitutive expression of the chimeric kinase leads to downstream activation of proteins such as SRC [2], PLCG and STAT3/5 [3], which promote tumor progression. SCLL is an aggressive disease and there are relatively few survivors even following bone marrow transplantation.

Genomic analysis has identified gene expression changes associated with the progression of SCLL to AML [4–6] as well as FGFR1-regulated changes in expression of microRNAs (miRNAs) [7–9]. Several of these miRNAs promote disease progression following their direct upregulation by FGFR1 [7]. The miR17/92 family, for example, led to increased cell proliferation through regulation of PTEN and CDKN1A [7] and miR339 caused inactivation of apoptosis suppressors BAX and BCL2L11 [8]. In the same analysis, a series of miRNAs were downregulated as a result of FGFR1 expression, which included miR150 [9]. miR150 expression, which normally suppresses the MYB oncogene, was silenced in SCLL through FGFR1-regulated DNMT1-directed promoter methylation, leading to derepression of MYB expression and increased cell proliferation [9]. These observations suggested an alternative mechanism of promoting leukemogenesis directed by FGFR1 activation beyond kinase activation of cellular proteins. As part of this analysis, miR-146b-5p showed reduced expression in SCLL cells. miR-146b-5p has previously been implicated in leukemia development and targets genes such as IRAK1 [10], which has been implicated in a wide variety of cancer phenotypes as well as influencing the immune response [11, 12].

IRAK1 is a member of a structurally and functionally related four-gene family of serine/threonine kinases and is activated by IL-1 [13]. The IRAK family members are critical components of the innate immune system and mediate signals downstream of various pathogen- and cytokine-responsive receptors such as IL-1 and lipopolysaccharide (LPS) through the IL-1 receptor (IL1R) and other toll-like receptors (TLR), respectively. Activation of IL1R and TLR recruits MYD88, resulting in activation of IRAK4 and IRAK1. Activated IRAK1/4 proteins subsequently activate TRAF6-mediated NF-κ B and p38MAPK [12, 13]. We now show that FGFR1 fusion kinase-mediated activation of IRAK1 in SCLL cells leads to increased accumulation of myeloid-derived suppressor cells (MDSCs) in vivo with a concomitant suppression of CD4/CD8 T-cells promoting an immune suppressive microenvironment leading to leukemia development. This phenotype is accompanied by induction of IFN-γ production in the leukemia cells and transplantation of control SCLL cells into IFNGR1 null mice shows impaired MDSC induction and mitigated leukemogenesis. Thus, IRAK1 appears to represent a switch in SCLL cells that modulates immune surveillance.

## Materials and methods

### Cell and molecular studies

All cells were maintained in RPMI 1640 culture medium supplemented with 10% fetal calf serum. Murine cell lines were derived in-house [4, 6, 14] and constantly monitored for the expression of their definitive chimeric kinases. Human KG1 cells were obtained from ATCC and similarly charcterized for the expression of the FGFR1OP2-FGFR1 fusion kinase. Cell proliferation was assessed using Trypan blue exclusion assays over a time course and cell viability was measured using the CellTitre Glow assay according to the manufacturer’s instructions (Promega). Western blot, genomic DNA preparation, plasmid transfection, quantitative RT-PCR, miR146b overexpression, cell cycle and cell apoptosis assays followed standard procedures that have been described extensively previously [9, 15]. Antibodies used for western blotting (dilution 1:1000): β-Actin (Cell signaling, #5125), IRAK1 (Cell signaling, # 4504), p-IRAK1 (Sigma-Aldrich, SAB4504246), IFN-γ (Abclonal, # A12450), CXCL9 (Abclonal, # A19135), p-AKT (Cell Signaling, # 9272), AKT (Cell Signaling, # 9271), p- p38α (Cell Signaling, # 9211), p38α (Cell Signaling, # 9218), p-Stat3 (Cell Signaling, # 9134), Stat3 (Cell Signaling, # 4904).

### T-cell and MDSC function assays

For the T-cell killing assay, spleen-derived CD4+ and CD8+ T cell were isolated using the EasySep™ Mouse CD4+ T Cell Isolation Kit and CD8+ T Cell Isolation Kit (STEMCELL Technologies Inc) from the IRAK1 KO cell engrafted leukemia free mice, respectively, and co-cultured with IRAK1 KO leukemia cells at different ratios in RPMI with 10% FBS for 48 h. Co-cultured leukemia cells were stained with Annexin V, with exclusion of immune cells by gating on the CD4-CD8- population [16]. For the MDSC suppression assay, MDSCs were isolated using the EasySep™ Mouse MDSC (CD11b + Gr1+) Isolation Kit (STEMCELL Technologies Inc.) from the MC cell engrafted mice with leukemia. The isolated CD4+ or CD8+ T cells were first stained using the CellTrace™ CFSE Cell Proliferation Kit (Thermo Fisher Scientific) and then co-cultured with MDSC for 6 days at varying ratios in the presence of anti-CD3/anti-CD28 Dynabeads (Gibco, #11456D). T cell proliferation was then analyzed using flow cytometry for CFSE by gating on the CD4+ or CD8+ population [17]. For the CD4+ T cell polarization assay, activation of T-cells was achieved using the BioLegend T-cell activation cocktail (Biolegend, #423301) according to the manufacturer’s instructions. Th1 type cytokines Ifn-γ (Biolegend, #505806) and Tnf-α (Biolegend, #506324), Th2 cytokines IL-4 (Biolegend, #504106) and IL-13 (Invitrogen, #25–7133-82), or Th17 cytokines IL-17A (Biolegend, #506916) and IL-22 (Invitrogen, #46–7221-82). All antibodies for flow cytometry were diluted at 1:100.

### CRISPR/Cas9 knockout

For locus-specific deletion of IRAK1 in BBC2 cells, we designed two guide sgRNAs of 20 nucleotides (nt) targeting the 5′ and 3′ flanking regions of the gene (sgRNA1: 5′-CGCGGCCTTCCGCCACACGG-3′; sgRNA2: 5′-GAGTGTCACTCAAGATTGCA-3′), using CRISPR Targets Track on Genome Browser [18]. Two additional sgRNAs, sgRNA3 5′- TCGTGCGACACCCGGCCGGG-3′ and sgRNA4 5′- AGCCAGCATCACTAGCAGGG-3′ were used for further validation in the independent ZNF112 cell line. The mock control clones were generated with the non-targeting sgRNA sequence 5′-AAAUGUGAGAUCAGAGUAAU-3′ (Thermo Fisher Scientific). Virus packing and single clone selection was performed as described previously [19].

### Detection of secreted proteins

Cells were treated with IL-1β or LPS and the supernatant collected. Bead-based immunoassays were used to screen for candidate cytokines or chemokines using the LEGENDplex™ multiplex assay (BioLegend) for the mouse inflammation panel including TNF-α, IFN-γ, IL-1α, IL-1β, IL-6, IL-10, IL-17A, IL-12p70, GM-CSF, IL-23, IFN-β, MCP-1, IL-27, and a customerized panel including IFN-γ, IFN-α, IFN-β, CCL11 (Eotaxin), CCL2 (MCP-1), CCL20 (MIP-3α), CCL3 (MIP-1α), CCL5 (RANTES), CXCL1 (KC), CXCL10 (IP-10), CXCL12 (SDF-1), CXCL9 (MIG). The Mouse IFN-γ Quantikine ELISA Kit (R&D Systems) was used for further validation.

### In vivo studies

Unless specified, 1 × 10^4^ cells were injected into the tail veins of 6–8-week-old female BALB/c mice and after one week, peripheral blood (PB) was withdrawn for immune monitoring or drug treatment. Engraftment ratios of GFP+ leukemia cells in PB were determined as described previously [20]. CD4-APC (Biolegend, #100412), CD8α-PE/Cy7 (Biolegend, #100722), Ly6C-PE (Biolegend, #128008), CD11b-PerCP/Cy5.5 (Biolegend, #101228), Ly6G-APC/Cy7 (Biolegend, #127624), CD314 (NKG2D)-AF555 (Bioss, # BS-0938R-A555), CD49b PerCP/Cy5.5 (Biolegend, #108916), CD3-PerCP/Cyanine5.5 (Biolegend, #100218), TCR γ/δ-APC/Cy7 (Biolegend, #118143), Foxp3-PE (Biolegend, #126404), CD68-BV421 (Biolegend, #137017), F4/80-PE (Biolegend, #157304) flow antibodies were used for monitoring immune cell composition, and PD-L1-PE/Cy7 (Biolegend, #124313), PD-1-PE (Biolegend, #135205), Ifngr1-PE (Invitrogen, #12–1191-82) flow antibodies were used for detecting surface marker expression in specific cell populations. Characterization of memory and effector T cells was performed using the Biolegend mouse naïve/memory T cell ID panel (Biolegend, #147501), with the inclusion of a CD8α-PE/Cy7 antibody. For CD4+/CD8+ T-cell depletion, purified anti-CD4 antibody (Bio X Cell, clone GK1.5) or anti-CD8β (Bio X Cell, clone 53–5.8) or non-immune rat IgG (ICN Pharmaceuticals, Aurora, OH) were administrated 500 μg/mouse i.p [16]., weekly for 3 weeks, with the first dose introduced 2 days before the IV injection of 1 × 10^4^ BBC2 IRAK1 KO cells. For MDSC depletion, purified anti-mouse Ly6G/Ly6C (Gr-1) antibody (Bio X Cell, clone RB6-8C5) or anti-mouse Ly6G (Bio X Cell, clone 1A8) or non-immune rat IgG (ICN Pharmaceuticals, Aurora, OH) were administrated 200 μg/mouse i.p [21]., three times a week for 4 weeks, with the first dose introduced 2 days after the IV injection of 1 × 10^4^ BBC2 MC cells. For pacritinib treatment, the randomly grouped xenografted mice were treated from days 7–12 with either vehicle or drug at 150 mg/kg twice daily by oral gavage [22]. Mice were sacrificed in the presence of advanced disease.

### Statistical analyses

All statistical analysis was performed using the Student’s T test to determine whether the means of two data sets are significantly different from each other. *p = < 0.01, **p ≤ 0.001, ***p ≤ 0.0001, ****p = 0.00001. ns = not significant. Error bars represent standard deviation. Unless otherwise stated, in vitro assays were repeated in triplicate and in vivo experiments involved cohorts of mice where *N* = 5. Kaplan-Meier statistical approaches were used to analyses differences in survival between different cohorts of mice.

## Results

### miRNA-146b-5p expression is suppressed in SCLL

**Fig. 1:**
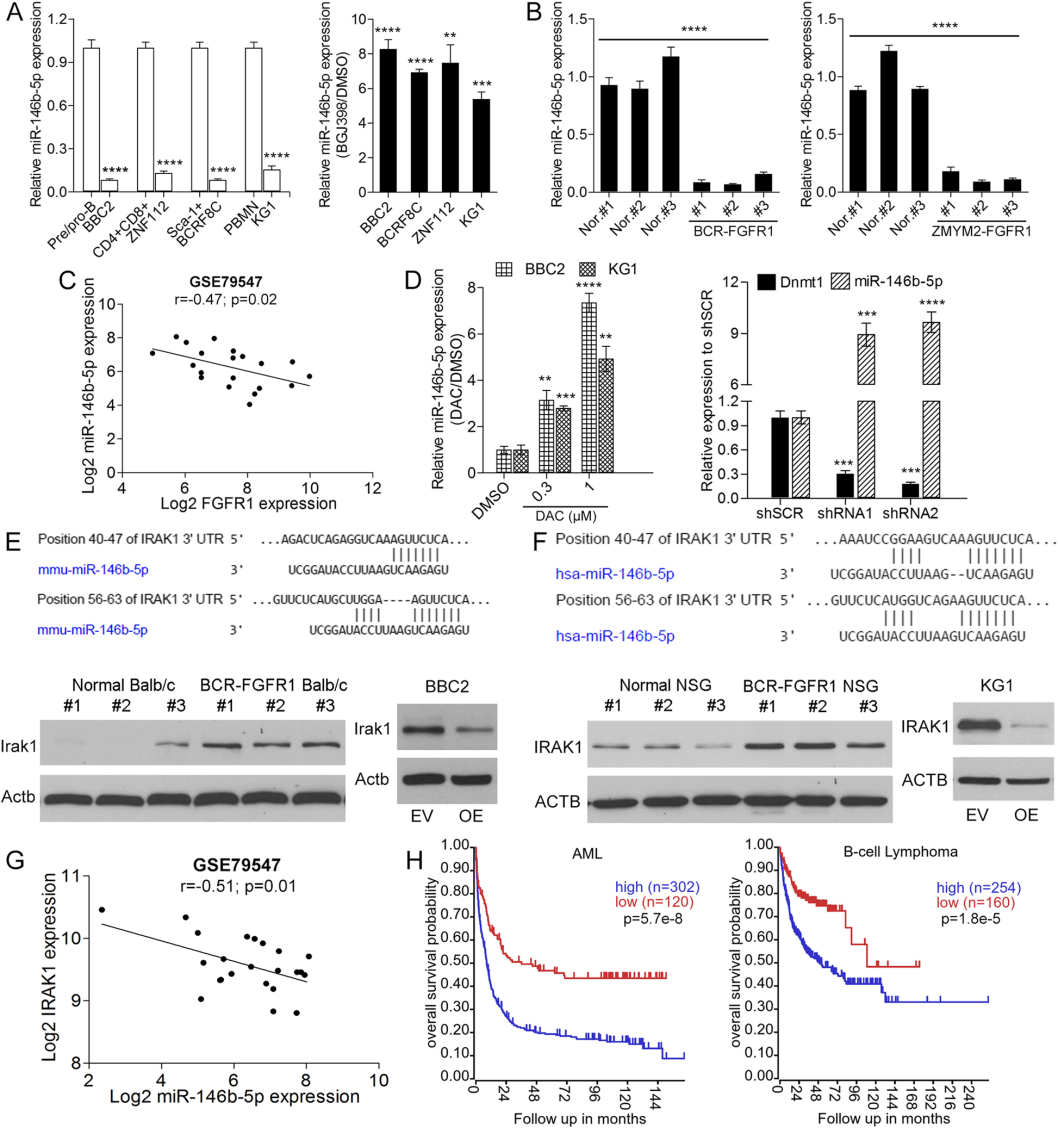
Relative expression levels (**A**, left) of miR-146b-5p in BBC2 cells relative to normal pre/pro-B cells, ZNF112 relative to normal CD4 + CD8 + cells, BCRF8C relative to normal Sca1+ cells and KG1 relative to peripheral blood mononuclear cells (*N* = 3 in all cases) shows consistent downregulation. When the same cell lines are treated with the BGJ398 FGFR1 inhibitor (*N* = 3) miR-146-5p levels increase compared with DMSO treated cells (**A**, right). Analysis of primary leukemic cells from a BCR-FGFR1 SCLL mouse model (N = 3) shows reduced levels of miR-146b-5p compared with normal BALB/c spleen cells (**B**, left). The same reduced levels of miR-146b-5p are seen in primary leukemic cells from the ZMYM2-FGFR1 mouse model (N = 3) of SCLL (**B**, right). Analysis of expression levels in primary human GSE79547 B-ALL samples (*N* = 20) shows a reverse correlation between miR-146b-5p and FGFR1 expression levels (**C**). When BBC2 and KG1 cells (N = 3) are treated with the 5-aza-2’deoxycytidine (DAC) methylation inhibitor (**D**, left) there is a dose-dependent increase in miR-146b-5p expression levels compared with DMSO treated controls. In BBC2 cells in which Dnmt1 is knocked down using two different shRNAs (**D**, right) there is ~ 10 fold increase in miR-146b-5p expression compared with BBC2 cells treated with a scrambled control (shSCR). Location of the miR-146b-5p target sites within the murine (**E**, above) and human (**F**, above) IRAK1 mRNA. Western blot analysis of IRAK1 expression in primary leukemic cells from the syngeneic BCR-FGFR1 model of SCLL shows significantly increased IRAK1 expression levels (**E**, left below). In BBC2 cells forced to overexpress (OE) miR-146b-5p (**E**, right below), there is also a highly significant decrease in IRAK1 expression levels compared with cells expressing the empty vector (EV). IRAK1 expression levels are also increased in leukemic cells from a human SCLL model for BCR-FGFR1 transformed CD34+ cells propagated in immunocompromised (NSG) mice (**F**, left below). In human KG1 cells forced to overexpress miR-146b-5p, IRAK1 level is reduced (**F**, right below). Analysis of the GSE79547 expression data set from B-ALL patients shows an inverse correlation between IRAK1 expression levels and expression of miR-146b-5p (**G**). Kaplan-Meyer analysis of an AML cohort [25] (*N* = 422) demonstrates a highly significant decrease in overall survival in patients showing high level expression of IRAK1 compared with those showing low level expression (**H**, left). A similar, although slightly less significant relationship between IRAK1 expression and survival is also observed (**H**, right) in a B-Cell lymphoma (*N* = 414) data set [26]. In each case the cut off is determined using the SCAN algorithm within the R2 Gemome Analysis and Visualization Platform. ** *p* < 0.01, *** *p* < 0.001, **** *p* < 0.0001

BBC2 cells express BCR-FGFR1 and lead to rapid (< 20 days) onset of B-cell leukemia/lymphoma [14]. Analysis of miRNA levels in BBC2 cells suggested a downregulation of miR-146b-5p [7] compared with normal counterpart cells. RT-PCR analysis of the murine ZNF112 [4], BCRF8C [6] and human KG1 [23, 24] SCLL cell lines (Fig. 1A), showed consistently reduced expression of miR-146b-5p in all cases. When treated with the BGJ398 FGFR1 inhibitor [27], miR-146b-5p expression levels increased in all cell lines (Fig. 1A). Primary leukemic bone marrow cells from BCR-FGFR1 or ZMYM2-FGFR1 murine transduction models (Fig. 1B) also show miR-146b-5p downregulation. When miR-146b-5p expression levels were analyzed in the GSE79547 human B-cell precursor acute lymphoblastic leukemia data set [28], there was a significant inverse correlation between miR-146b-5p expression and FGFR1 expression (Fig. 1C). Previous studies [9] suggested that FGFR1-mediated upregulation of Dnmt1 led to miRNA promoter methylation and, when either BBC2 or KG1 SCLL cells (Fig. 1D) were treated with 5′-aza-deoxycytidine, a dose-dependent increase in miR-146b-5p expression levels was seen. Dnmt1 is the dominant DNA methyltransferase in SCLL cells [9] and knockdown of Dnmt1 in BBC2 cells using shRNAs showed an ~ 10-fold increase in miR-146b-5p expression (Fig. 1D), further confirming methylation-mediated downregulation.

### miR-146b-5p regulates Irak1 in SCLL cells

IRAK1 is a target of miR-146b-5p [10] and the target sequences in the 3′ UTR are highly conserved in different mammalian species (Supplementary Fig. 1). There are two tandem miR-146b-5p target sites in both mouse and human (Fig. 1E-F). Overexpression of miR-146b-5p in BBC2 and KG1 cells (Supplementary Fig. 1) led to a significant downregulation of IRAK1 (Fig. 1E-F). Primary SCLL cells from the BCR-FGFR1 driven model [14] also showed increased IRAK1 levels (Fig. 1E), consistent with low levels of miR-146b-5p expression in SCLL (Fig. 1A). Similarly, BCR-FGFR1 transformed human CD34+ cells, propagated in immunocompromised mice [29] also showed increased IRAK1 levels (Fig. 1F). Analysis of the GSE 79547 B-ALL data set [28] also shows an anti-correlation between IRAK1 and miR-146b-5p expression (Fig. 1G). Survival analysis for human AML and B-lymphomas [25, 26], shows highly significant decreases when IRAK1 is expressed at high levels (Fig. 1H). Thus, activation of IRAK1 appears to promote aggressive development of SCLL.

### Deletion of IRAK1 prevents tumor development by SCLL cells

**Fig. 2:**
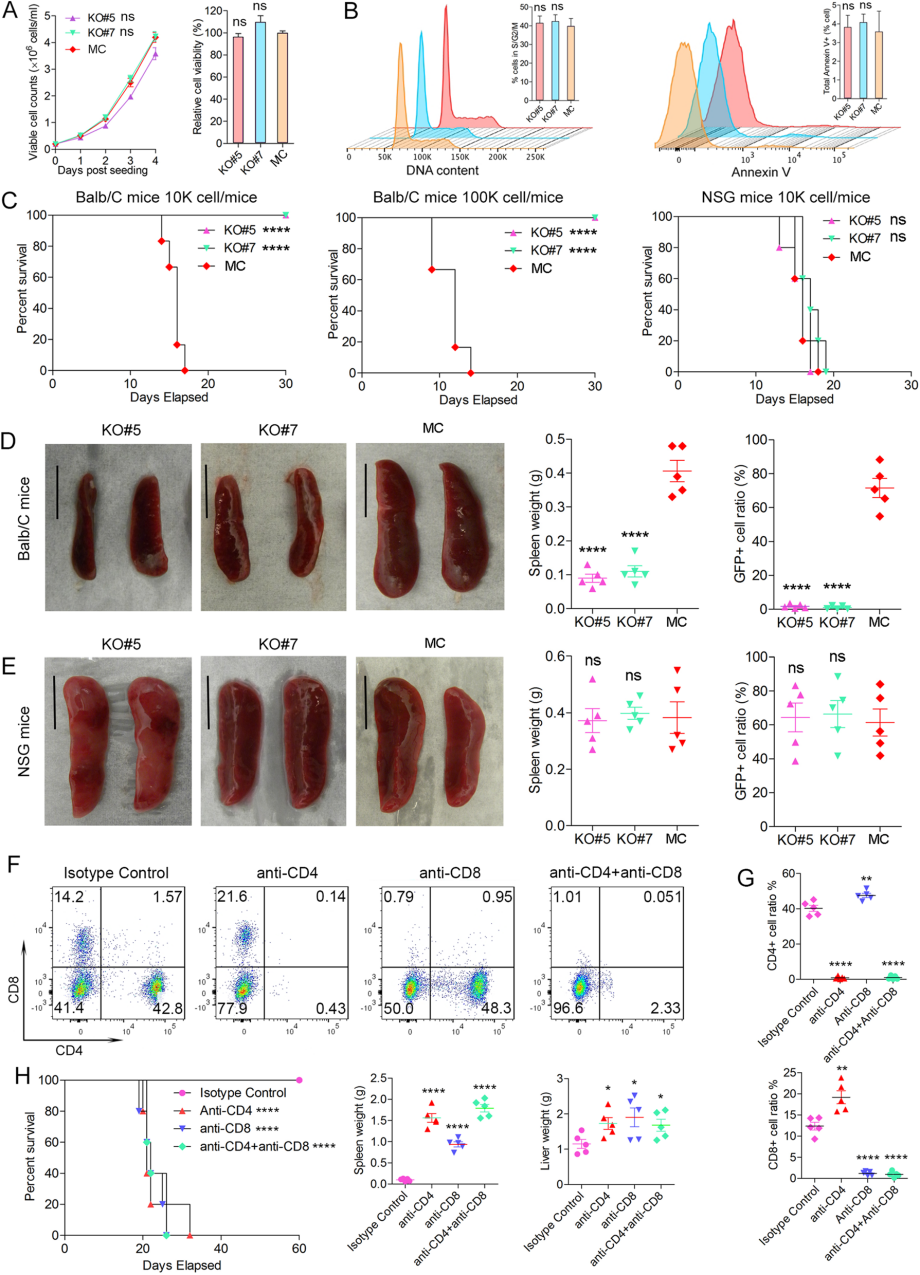
Analysis of relative cell number increase using the trypan blue exclusion assay (*N* = 3) over 4 days in the two Irak1 KO clones shows no difference compared with the mock control (MC) BBC2 cells (**A**, left). Analysis of cell viability, relative to that seen in the MC cells, after 4 days (N = 3), using the CellTiter Glow assay (**A**, right), shows no difference between the KO and MC cells. Flow cytometric analysis (**B**, left) shows no difference in the distribution of cells in the S/G2/M phases of the cell cycle when the two KO clones were compared individually with the MC cells (N = 3). There is also no difference in levels of apoptosis as determined by annexin V staining (**B**, right). Analysis of BALB/c mice xenografted with either 10,000 (**C**, left) or 100,000 (**C**, center) cells (*N* = 6) from KO clones #5 and #7 and MC cells that show survival times of 14–17 days in mice xenografted with the MC BBC2 cells. In contrast, mice xenografted with the KO clones did not develop leukemia during the observation period up to 120 days regardless of inoculated tumor cell burden. When 10,000 cells from the MC and KO lines were engrafted into immunocompromized NSG mice (**C**, right) however, all mice (*N* = 5) developed disease and died within 13–19 days. The survival data shown in (**C**) was reflected in a significant reduction in spleen size and the proportion of GFP+ cells in the spleens from mice xenografted with the KO clones when compared individually with mice xenografted with the MC cells at autopsy (**D**). Spleen size in the NSG mice (**E**) was enlarged in all cohorts and levels of GFP+ cells were equally high. Treatment of mice (*N* = 5) with an anti-CD4 or anti-CD8 antibody shows a significant reduction in CD4+ or CD8+ cells, respectively (**F** and **G**) when compared individually with the isotype control. Conbination treatment with anti-CD4/CD8 antibodies led to significant loss of both cell types as shown in **F** and **G**. When IRAK KO #7 cells are xenografted into BALB/c mice treated with the various antibodies to deplete T-cells, while the isotype treated mice did not develop leulemia, mice experiencing depletion of CD4 or CD8 or both types of cells showed robust development of leukemia when each is compared with the isotype control (**H**, left). The development of leukemia was paralleled with increased spleen and liver weight (**H**, right). ns = not significant, * *p* < 0.05, ** *p* < 0.01, **** *p* < 0.0001

CRISPR/Cas9 deletion of exons 1–12 in BBC2 cells (Supplementary Fig. 2A) generated individual clones showing homozygous loss of IRAK1 (Supplementary Fig. 2B-C). Clones #5 and #7 were randomly selected for further analysis. A mock control (MC) was generated using a scrambled sgRNA to exclude any clonal selection effect or potential immune response from constitutive expression of Cas9. Loss of IRAK1 had no significant effect on cell growth or viability (Fig. 2A) and cell cycle progression in vitro was also unaffected (Fig. 2B, left). Following annexin V staining no noticeable differences in cell apoptosis was observed either (Fig. 2B, right). It seems, therefore, that IRAK1 may more specifically affect leukemia progression through a mechanism distinct from cell proliferation/viability.

To study disease progression in vivo, BBC2 MC and IRAK1 KO cells were engrafted into unirradiated, syngeneic BALB/c hosts and, while the MC cells displayed rapid disease progression leading to death within 14–17 days (Fig. 2C), all mice engrafted with KO clones #5 and #7, showed no symptoms of disease during the 120-day observation period (Fig. 2C). The engraftment ratio, as determined by the levels of GFP+ cells in the peripheral blood from the various cohorts, also reflected the disease load in these mice (Fig. 2D). A parallel engraftment in immunocompromised NSG mice however, showed no difference in disease progression between the MC BBC2 and the KO clones (Fig. 2C, E), suggesting IRAK1 may suppress the host immune response.

To determine whether IRAK1 has a broader influence on different leukemia subtypes, we used CRISPR/Cas9 to knock out IRAK1 in ZMYM2-FGFR1 expressing ZNF112 cells [4], which predominantly show a T-cell ALL immunophenotype. Four knockout clones, #9 #13, #15 and #16, were identified with homozygous deletion of IRAK1 (Supplementary Fig. 2D-E). Xenograft studies showed that clones #9 and #13 did not develop leukemia in immunocompetent BALB/c mice, which is also reflected in the relative spleen weights in the different cohorts (Supplementary Fig. 2F). When the same cells were xenografted into NSG mice (Supplementary Fig. 2G), all three cell lines showed rapid onset of leukemogenesis, suggesting IRAK1 plays a similar role in immune evasion in both BCR-FGFR1 and ZMYM2-FGFR1 driven SCLL models.

### IRAK1 KO leukemia cell clearance is mediated by CD4+/CD8+ T cells

Because IRAK1 KO cells generate leukemia in immune-deficient NSG mice but not in immune-competent syngeneic hosts, the immune system was implicated in playing a critical role in eradication of the IRAK1 KO cells. To examine wethere innate immunity plays a role in this leukemia clearance, we first performed flow analysis of the NK cell and γ/δ T cell population in our model. NK cell populations in the peripheral blood using CD314 and CD49b (Supplemental Fig. 3A-B) showed no differences between KO#7 engrafted mice and naïve mice. The same was true for γ/δ T-cells using the TCR γ/δ and CD3 markers. Then, to determine whether this is mediated directly by α/β T-cell mediated adoptive immunity, we used antibodies to deplete CD4+ and CD8+ cells in immune-competent BALB/c host mice (Fig. 2F). Compared with isotype controls, CD4+ or CD8+ cells were almost completely depleted with the anti-CD4 or anti-CD8 antibodies, respectively, similar to effects observed in the simultaneous depletion of both CD4 and CD8 expressing cells using both antibodies (Fig. 2G). When IRAK1 KO#7 cells were inoculated into the four different groups of mice, disease did not develop in isotype treated mice but leukemia developed in mice that were depleted either individually for CD4 or CD8 or combined CD4/CD8 cells (Fig. 2H). The disease development in the individual cohorts was reflected in spleen and liver weights (Fig. 2H). These data are consistent with idea that either the loss of helper function of CD4+ cells for CD8+ cells or failure in realization of the CD8+ killer effects in those mice engrafted with IRAK1 KO cells will allow the emergence of leukemia, which confirms a direct involvement of T cells in clearance of IRAK1 KO cells, further supporting a function for IRAK1 in modulating the immune system.

**Fig. 3:**
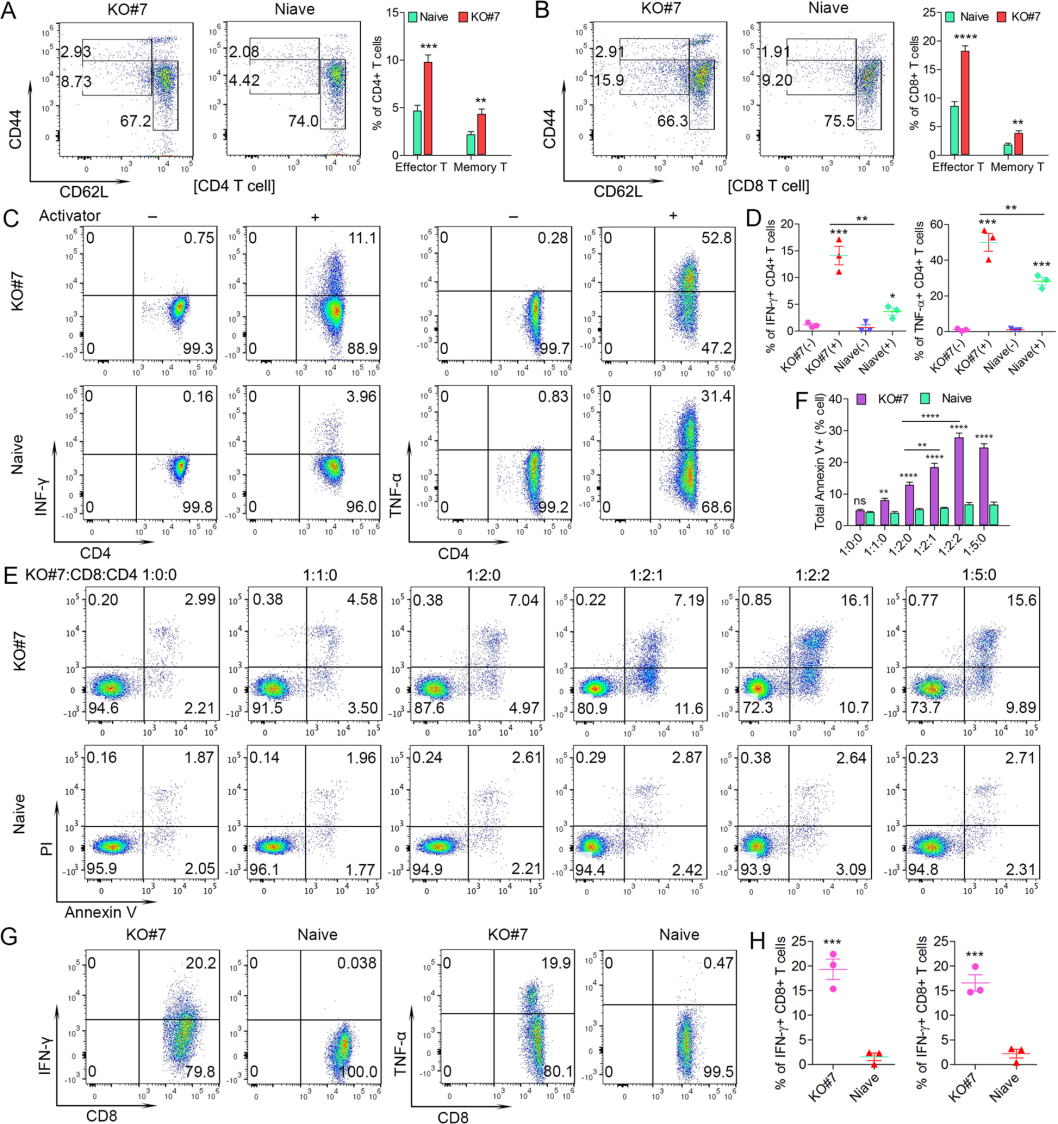
Representative flow cytometric analysis of CD62L and CD44 expression in CD4+ (**A**) or CD8+ (**B**) T cells from peripheral blood of mice (N = 5) xenografted with the KO#7 clone compared with age matched naïve mice at the time of sacrifice shows an increase in activated effector T-cells (CD44^int^CD62L^lo^) and memory T-cells (CD44^hi^CD62L^lo^) only in the mice xenografted with KO#7 cells (**A** and **B**). Following in vitro activation using the BioLegend cell activator cocktail (*N* = 3), there is a significant increase in levels of the Ifn-γ and Tnf-α cytokines in CD4+ cells from the spleens compared with untreated, age-matched naive control mice (**C** and **D**). Tumor killing assays (N = 3) in vitro demonstrate proportionally increased annexin V+ staining in BBC2 KO#7 cells when cultured with increasing levels of CD8+ cells derived from KO#7 xenografted mice (**E** and **F**) but not when co-cultured with CD8+ cells from naive mice. In addition, the inclusion of CD4+ T helper cells leads to increasesd annexin V+ staining in BBC2 KO#7 cells (**E** and **F**). These tumor-killing CD8+ cells derived from mice engrafted with KO#7 cells, compared with cells from wild type mice, also showed increased levels of Ifng and TnfA cytokines (**G** and **H**). ns = not significant, * *p* < 0.05, ** *p* < 0.01, *** *p* < 0.001, **** *p* < 0.0001

Since the observation indicates that CD4+ and CD8+ cells are responsible for the rejection of IRAK1 KO SCLL cells in syngeneic hosts, we investigated the status of these T cells in these leukemia free mice. Using CD44 and CD62L as markers of T cells for different stages (Fig. 3A and B), there was a clear and significant increase in the CD44^int^CD62L^lo^ effector T-cells and CD44^hi^CD62L^lo^ memory T-cells in mice xenografted with KO#7 cells compared with age-matched naïve mice (Fig. 3A and B). To further examine the activation of CD4+ T-cells in the IRAK1 null leukemia model, we performed CD4 cell polarization analysis with splenocytes isolated from IRAK1 KO cell-engrafted, tumor free mice. Compared with the naïve control mice, there is an increase in Ifn-γ and Tnf-α expressing CD4+ T cells after stimulation with a cell-activator cocktail, indicating a differentiation into Th1 cells (Fig. 3C-D). There were no differences in the Th2 cytokines, IL-4 and IL-13, or Th17 cytokines, IL-17A and IL-22, between CD4 T cells from IRAK1 KO cell-engrafted mice and naïve control mice (Supplemental Fig. 3C-F).

The tumor clearance activity of CD8+ T-cells from IRAK1 KO cell-engrafted mice was further examined using tumor cell killing assays. The CD8+ T-cell population was recovered from tumor-free mice engrafted with the IRAK1 KO #7 cells and naive BALB/C mice and then co-cultured in vitro with BBC2 KO#7 cells at different ratios (Fig. 3E). Annexin V staining showed significant increases in apoptosis levels in the BBC2 KO#7 leukemic cells, which was proportional to the number of CD8+ cells included in the assay, compared with the CD8+ cells isolated from naive mice (Fig. 3E and F). To further validate the activity of CD4+ T helper cells in tumor clearance, we also introduced CD4+ T cells into this tumor killing assay which further increased the tumor killing activity of CD8+ cells (Fig. 3F). These results directly demonstrate the ability of the CD8+ cells that are derived from mice xenografted with KO#7 cells to kill the BBC2 KO#7 cells, and CD4+ T cells can enhance this tumor clearance activity. When expression of T-cell activity markers Ifn-γ and Tnf-α was analysed in these CD8+ cells, both cytokines showed increased levels only in the cells isolated from the KO#7 engrafted mice (Fig. 3G), consistent with the tumor killing activity.

Together, these experiments demonstrate that loss of IRAK1 expression in the tumor cells leads to immunity related tumor clearance where both the activated CD4+ and CD8+ T-cells are directly responsible for suppressing leukemia development.

### IRAK1 expression promotes MDSC induction and subsequent suppression of CD4+/CD8+ cells

**Fig. 4:**
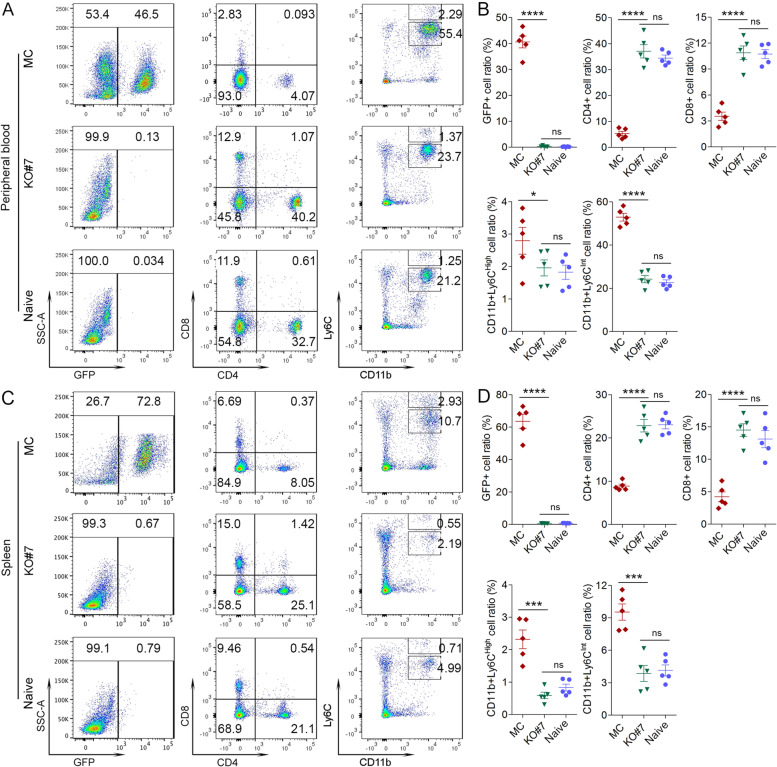
Analysis of peripheral blood shows disease development in mice carrying the MC cells but not in KO#7 as measured by the levels of GFP+ cell numbers. There is a highly significant decrease in levels of CD4 + (85.58%) and CD8+ (67.43%) cells in the mice carrying the MC cells compared with the mice carrying KO clone #7, which shows no differences with the naïve normal control BALB/c mice without engraftment (**A**). A more more than 100% increase in the levels of CD11b+/Ly6C+ MDSCs is seen in mice injected with the MC BBC2 cells (**B**). At this stage (**C**) the disease has progressed significantly as shown by more than 60% GFP+ cells in the spleens from mice (N = 5) carrying the MC cells but there are virtually no GFP+ cells in the spleens from mice inoculated with KO#7 cells which is comparable with the naïve mice. The percentage of MDSC and CD4+/CD8+ cells in the spleens from the two cohorts show the same trend seen in the PB (**D**). The proportions of MDSC and CD4+/CD8+ cells in the PB of mice carrying the KO#7 cells are virtually identical to those seen in PB from naïve BALB/c mice. * *p* = < 0.01, *** *p* = < 0.0001, **** *p* = < 0.00001

Our next question was why IRAK1-expressing, mock control leukemia cells survived and cause disease in hosts with a competent immune system. It has been reported that the immune system can profoundly influence tumor development and progression through orchestrating the suppressive effects of MDSC on CD4+/CD8+ cell activity [30]. MDSCs are categorized as either CD11b−/Ly6C^hi^ monocytic MDSC (mMDSC) or CD11b+/Ly6C^int^ granulocytic MDSC (gMDSC) [16, 31]. Flow cytometry analysis of peripheral blood (PB) from mice ~ 16 days after being xenografted with BBC2 MC cells, shows an advanced disease with 46.5% GFP+ leukemic cells compared with virtually no GFP+ cells in mice xenografted with the KO#7 cells (Fig. 4A). At this stage CD11b+/Ly6C^int^ levels in the PB of unirradiated BALB/c mice showed a 118% increase in mice xenografted with the MC cells compared with mice xenografted with KO#7 cells (Fig. 4A-B). The same changes were observed (Fig. 4C-D) in the spleens, where there is also no evidence of GFP+ leukemic cells in the mice xenografted with the KO#7 cells, compared with more than 60% of GFP+ cells in mice inoculated with MC cells, and which was accompanied by significant increases of both the CD11b+/Ly6C^hi^ and CD11b+/Ly6C^int^ MDSCs. Since MDSCs are known to down regulate the T-cell receptor and reduce levels of T-cells [30, 31], analysis of PB and spleen samples from mice xenografted with the MC BBC2 cells showed significantly decreased CD4+ and CD8+ cells compared with mice carrying KO#7 cells, which is consistent with an inverse relationship with MDSC levels (Fig. 4). The same increase of MDSCs was seen in the bone marrow from these mice at the time of sacrifice (Supplementary Fig. 4A-B). The levels of MDSC and CD4+/CD8+ cells in the mice carrying the KO#7 cells are virtually identical to those seen in the relative cellular counterparts in normal BALB/c mice (Fig. 4 and Supplementary Fig. 4A-B). In addition, analysis of regulatory T cells (Tregs) and macrophages showed no significant differences between peripheral blood cells from mice engrafted with either the MC cells or KO#7 cells (Supplenetary Fig. 4 C-D), further confirming the critical role of MDSCs in immune suppression. Similarly, analysis of peripheral blood cells in mice xenografted with ZNF112 MC and IRAK1 KO#9 cells showed the same impaired MDSC induction as a result of IRAK1 knockout and a concomitant failure to suppress CD4+/CD8+ T-cells (Supplementary Fig. 4E-F). Thus, in different subtypes of SCLL, IRAK1 promotes accumulation of MDSCs with a consequential suppression of cytotoxic T-cells, allowing development of SCLL, supporting the hypothesis that IRAK1 expressing SCLL cells escape immune recognition by inducing MDSCs, which suppress cytotoxic T-cell function, whereas loss of IRAK1 fails to suppress T-cell mediated leukemia cell clearance.

**Fig. 5:**
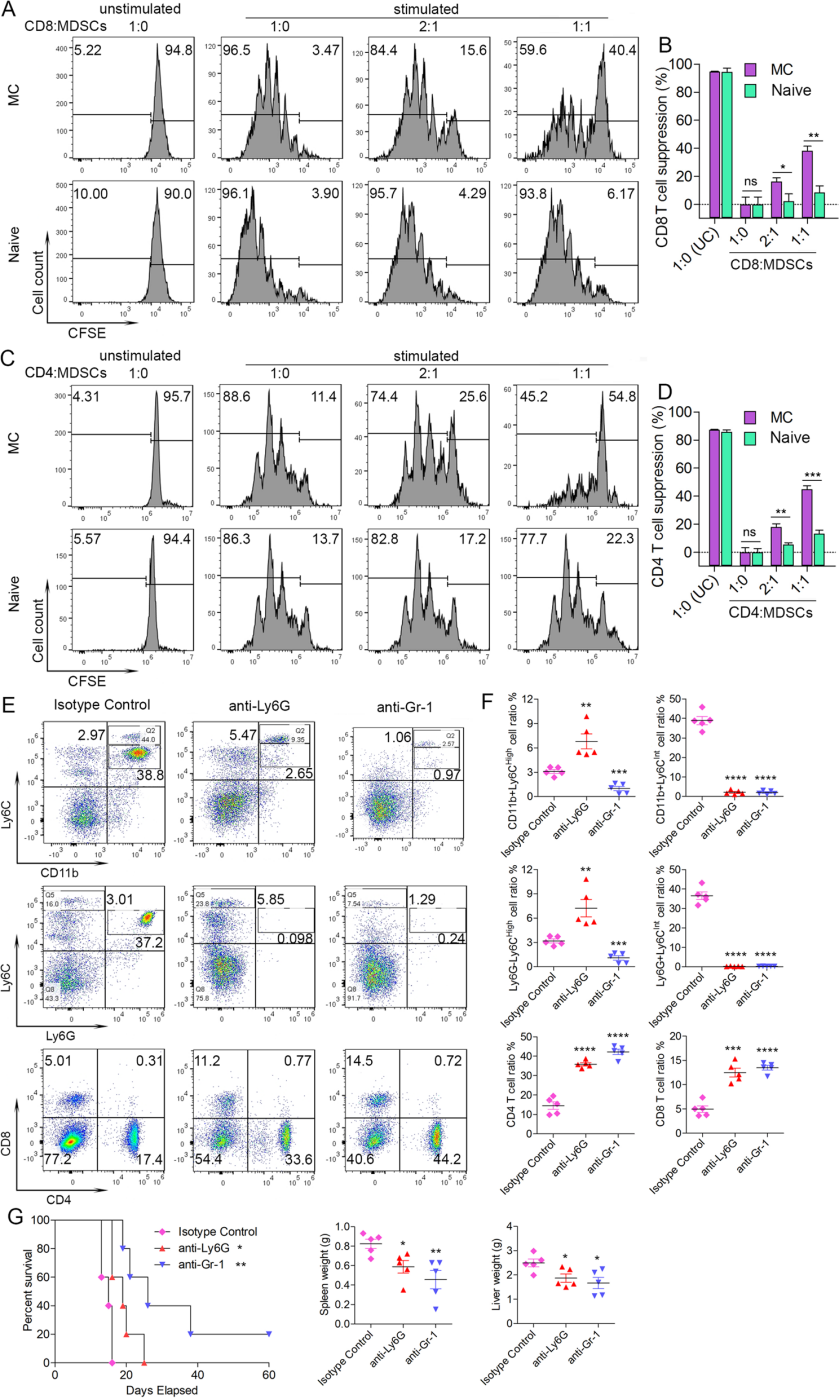
In the MDSC suppression assay, when CFSE stained CD8+ T-cells derived from mice xenografted with BBC2 KO#7 cells were co-cultured with MDSC obtained from mice xenografted with MC BBC2 cells at different concentrations (*N* = 3), there is a proportional reduction in proliferation compared with co-cultured MDSC obtained from naïve wild type mice (**A** and **B**). Unstimulated CD8+ cells do not proliferate whether derived from MC-engrafted or naïve mice. Stimulation of CD8+ cells proliferation in these cultures were performed with mouse CD3/CD28 Dynabeads (Gibco). CD8+ cells in the absence of MDSC show the same rates of proliferation for the MC and naïve cells. Suppression of cell proliferation in the stimulated cells was proportional to the relative level of MDSC (**B**). When the same experiments were performed using CD4+ cells derived from KO#7 cell engrafted mice, the same suppression were seen (**C** and **D**). In vivo MDSCs depletion assays were then performed using either anti-Ly6G or anti-Gr-1 antibodies (**E**-**G**). Compared with mice treated with the isotype control antibodies, treatment of mice with anti-Ly6G antibody leads to a significant depletion of CD11b + Ly6C^int^ gMDSC, with a passive increase of the CD11b + Ly6C^hi^ mMDSC, while treatment of anti-Gr-1 antibody leads to depletion in both the CD11b + Ly6C^int^ gMDSC and CD11b + Ly6C^hi^ mMDSC (**E** and **F**). In these analyses cell ratios for each immunophenotype are compared individually with those seen in the isotype control group. As a result of MDSC depletion, there is an increase in the CD4+ and CD8+ cell populations (**E** and **F** lower). Consistent with the increased presence of T cells, survival of KO#7 cells engrafted mice treated with the anti-Ly6C or anti-Gr-1 antibody was extended significantly, compared with the isotype treated mice, with one mouse of the anti-Gr1 treated mice remaining disease free at the end of observation (**G**). Disease progression was associated with decreases in liver and spleen weights compared with the isotype control (**G**). ns = not significant, * *p* < 0.05, ** *p* < 0.01, *** *p* < 0.001, **** *p* < 0.0001

Since our immune monitoring assay showed the increase in MDSCs is accompanied by reduced T cell populations, we first investigated whether these MDSCs execute a T cell suppressive function by performing MDSC-mediated T cell suppression assays [16, 32]. We isolated MDSCs, defined by the CD11b + Gr1+ phenotype, from the PB of BBC2 MC cell-engrafted mice and the normal counterparts from wild type naive mice. These cells were then co-cultured with CD8+ T cells from tumor-free mice engrafted with the IRAK1 KO cells in different ratios and proliferation was determined using flow cytometry (Fig. 5A). The unstimulated T-cells do not proliferate and the stimulated cells show robust proliferation in the presence of the anti-CD3/anti-CD28 Dynabeads. With a 2:1 and 1:1 ratio of CD8+/MDSCs, there is a significant suppression of T cell proliferation for MDSCs from BBC2 MC cell-engrafted mice compared with those cells from control naive mice, with ~ 40% T cell suppression in the 1:1 ratio co-culture (Fig. 5B). These results demonstrate the ability of MDSCs derived from the BBC2 MC cell-engrafted mice to suppress proliferation of tumor eradicating CD8+ cells, thus allowing tumor development. Given that CD4+ helper T cells are also involved in eradication of IRAK1 KO leukemia cells, we also analysed the ability of MDSC to suppress CD4+ T cells. When the same experiments were performed using CD4+ cells from tumor-free mice engrafted with the IRAK1 KO cells, we saw the same MDSC-mediated suppression of proliferation (Fig. 5C-D). This in vitro cell based assay clearly demonstrated the ability of MDSCs to suppress both CD4+ and CD8+ T cell activity.

To further confirm this MDSC mediated suppression of antitumor immunity in vivo, MDSC depletion experiments were performed in the BBC2 MC cell engraftment model using either the anti-Ly6G antibody or the anti-Ly6G/Ly6C (Gr-1) antibody. When immune competent BALB/c mice were treated with the anti-Ly6G antibody, there was an efficient depletion of CD11b + Ly6C^int^ gMDSC compared with the isotype treated controls, which were also positive for Ly6G marker (Fig. 5E and F). The same depletion of CD11b + Ly6C^int^ gMDSC was observed when the mice were treated with the anti-Gr-1 antibody simultaneously targeting Ly6C and Ly6G (Fig. 5E). Noticeably, only the anti-Gr-1 antibody could also deplete the CD11b + Ly6C^hi^ mMDSC, due to the absence of the Ly6G marker in the CD116 + Ly6C^hi^ mMDSC population (Fig. 5E and F). Most importantly, both single depletion of CD11b + Ly6C^int^ gMDSC with the anti-Ly6G antibody as well as double depletion of the CD11b + Ly6C^int^ gMDSC and CD11b + Ly6C^hi^ mMDSC with the anti-Gr-1 antibody, led to significantly increased levels of CD4+ and CD8+ cells, compared to the isotype control group (Fig. 5E-F). Consistent with the increase of T cells, mouse survival was significantly prolonged, with median survival of 15 days for the isotype group, 19 days for the anti-Ly6G antibody group and 26 days for the anti-Gr-1 antibody group (Fig. 5G). Even more impressively, one mouse in the anti-Gr-1 antibody cohort remained completely leukemia free at sacrifice after 60 days. The enhanced survial in the anti-Gr-1 antibody group indicates that the relatively rare CD11b + Ly6C^hi^ mMDSCs also contribute to immume suppression. The attenuated disease progression in the individual cohorts was also reflected in spleen and liver weights (Fig. 5G). These data support the idea that IRAK1 expressing leukemia cells induce the accumulation of MDSCs, which concomitantly suppress CD4+/8+ T-cell mediated leukemia clearance, while knockout of IRAK1 leads to failure in MDSC induction and subsequent immune suppression, resulting in T cell mediated clearance of the IRAK1 null leukemia cells.

### IRAK1 signaling activates interferon gamma production in SCLL cells

To investigate the mechanism of IRAK1 mediated immune evasion, pathways downstream of IRAK1 were examined in the BBC2 MC and IRAK1 KO cells. IL-1β, a potent pro-inflammatory cytokine facilitating host defense responses to infection, was used to activate IRAK1 [33]. A dose dependent activation of downstream signaling molecules such as pAKT and p38 was seen in the MC BBC2 cells but not in the IRAK1 KO clones (Supplmentary Fig. 5A). There was no effect on STAT3 activation in either cell system. Thus, IRAK1 mediated signaling pathways may be directly involved in the immune evasion process.

**Fig. 6:**
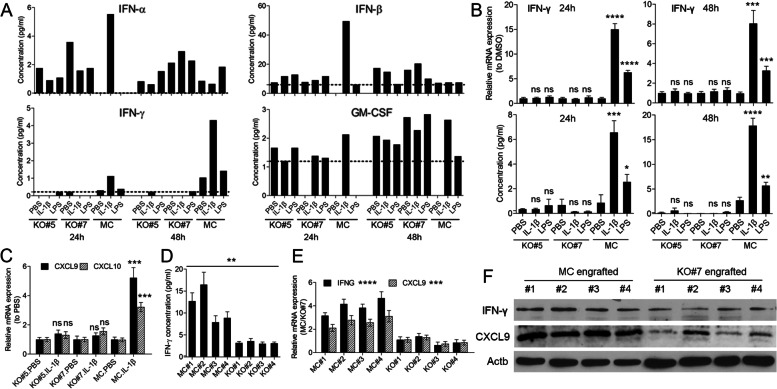
Profiling of cytokines in the supernatants from MC BBC2 and KO #7 cells cultured in vitro using LEGENDplex bead-based immunoassays over a 48-h period following stimulation with either LPS or IL-1β demonstrates specific and time dependent increases in IFN-γ only in the MC cells (**A**). Levels of IFN-α and IFN-β and GM-CSF do not show this specific activation. These data were confirmed at the mRNA expression level using RT-PCR (**B**, above) and protein level using ELISA (**B**, below) from independent treatments with either LPS or IL-1β (*N* = 3) compared individually with levels seen in the PBS treated cells. Analysis of the IFN-γ downstream targets CXCL9 and CXCL10 (**C**) shows increased levels only in the MC cells treated with IL-1β. In serum samples from mice engrafted with either MC or KO#7 cells (**D**), ELISA assays show significantly increased levels of IFN-γ in the MC cell engrafted mice compared with the KO cell engrafted group. Analysis of splenocytes from the same animals shows coincident increases in IFN-γ and CXCL9 mRNA expression in the MC cell engrafted mice only (**E**), which was confirmed at the protein level using western blotting (F; *N* = 4). ns = not significant, * *p* < 0.05, ** *p* < 0.01, *** *p* < 0.001, **** *p* < 0.0001

Since IRAK1 is known to induce a series of immune response regulatory cytokines and chemokines [12, 34], we used bead-based immunoassays to identify candidates directly affected by IRAK1 KO in SCLL. Secreted levels of a panel of 23 cytokines and chemokines related to immune regulation were analyzed in BBC2 MC and IRAK KO clones #5 and #7 following either LPS or IL-1β treatment (Supplementary Fig. 5B). Of the 16 proteins showing above detectable levels in the conditioned medium, only IFN-γ showed a consistent and time-dependent activation following stimulation with both agents in BBC2 MC cells, but not in the IRAK KO clones (Fig. 6A). In contrast, two other members of the interferon family, IFN-α and IFN-β, were not differentially affected and neither was GM-CSF, which has been implicated in MDSC induction [35]. This inducible increase in IFN-γ levels, due to activation of the IRAK1 pathway in the MC cells, was further validated at the mRNA level by RT-PCR (Fig. 6B). Analysis of the CXCL9 and CXCL10 genes, which are activated downstream of IFN-γ [36], showed upregulation in the MC BBC2 cells and not in the IRAK1 KO cells following stimulation with IL-1β (Fig. 6C). Consistent with the in vitro data, ELISA assays using serum derived from mice engrafted with either MC or KO#7 cells (*N* = 4) showed a significant increase in IFN-γ levels in the mice engrafted with MC cells compared with mice engrafted with KO#7 cells (Fig. 6D), and IFN-γ produced by the leukemia cells contributes 60–80% of total IFN-γ in the serum. In contrast, there is no detectable level of GM-CSF in the same leukemia bearing mice (data not shown). Analysis of mRNA expression levels in splenocytes from these same mice shows a concomitant increase in CXCL9 expression in the presence of increased IFN-γ expression only in the MC cell engrafted group (Fig. 6E). These observations are also supported by increased levels of IFN-γ and CXCL9 protein in splenocytes from MC engrafted mice compared with the KO #7 engrafted mice (Fig. 6F). Thus, we have shown that IFN-γ is an important downstream target of IRAK1 in SCLL cells, which may participate in IRAK1-mediated immune suppression processes.

### The PD-L1/PD-1 pathway is not involved in IRAK1-induced IFN-γ immune evasion

Knockout of IRAK1 appears to prevent IFN-γ production, suggesting its potential role in the development of immune evasion in SCLL models. IFN-γ is known to upregulate PD-L1, a ligand for the T-cell inhibitory receptor PD-1 [37, 38], which promotes T-cell exhaustion leading to suppression of anti-tumor immune responses. RT-PCR analysis of PD-L1/2 levels in the Irak1 KO and MC BBC2 cells following IL-1β stimulation (Supplementary Fig. 5C, above) showed increase expression of PD-L1 only in the MC cells. Levels of PD-L2 were consistently low even after IL-1β stimulation in either cell population. Flow cytometry analysis using a PD-L1-specific antibody (Supplementary Fig. 5C, below), revealed a > 4-fold increase in mean fluorescence intensity (MFI). These data implied that IRAK1 induced IFN-γ may upregulate PD-L1 expression in leukemic cells to facilitate the establishment of immune evasion through the PD-L1/PD-1 pathway. To examine this possibility, flow cytometry analysis of PD-1 expression in CD4+ and CD8+ cells from the MC BBC2 cell engrafted mice was performed where the same population levels of PD-1 positive CD4+ or CD8+ T cells was seen compared with the naïve controls (Supplementary Fig. 5D), suggesting that the PD-L1/PD-1 pathway is not responsible for immune evasion resulting from this IRAK1 activated IFN-γ signaling.

### IFN-γ is involved in the induction of MDSCs during establishment of immune evasion by SCLL cells

**Fig. 7:**
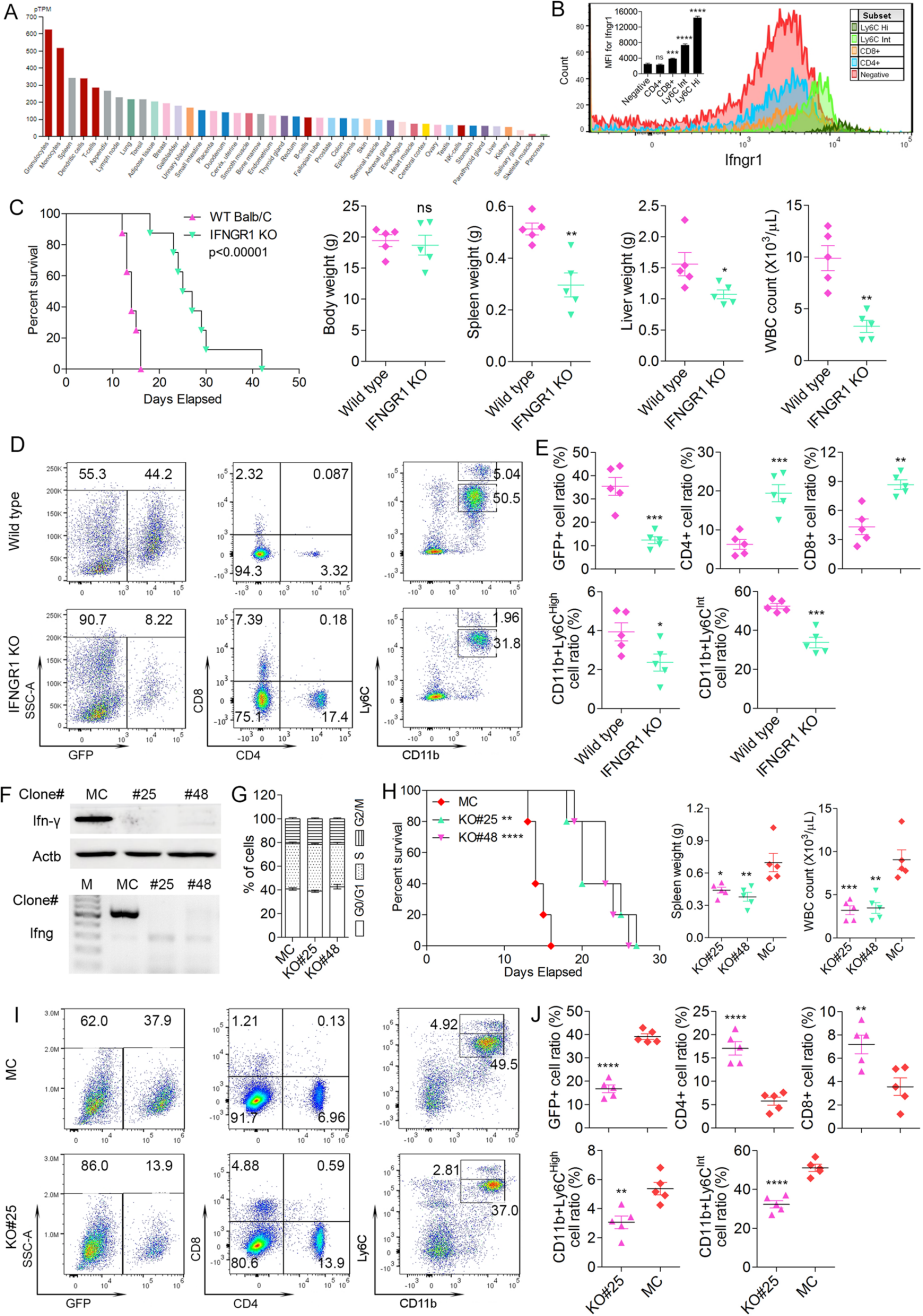
Relative expression levels (**A**) of the IFNGR1 in various cell types as reported in the Human Protein Atlas database. Flow cytometric analysis (N = 3) of IFNGR1 confirms its predominant presence in both CD11b + Ly6C^int^ and CD11b + Ly6C^hi^ myeloid cells (**B**) compared to CD4+/CD8+ cells and rest negative cells from wild type BALB/c mice. Kaplan-Meier analysis (*N* = 8) shows that when BBC2 cells were engrafted into host wild type BALB/c or Ifngr1 null mice (**C**), there is a significant increase in survival time in the Ifngr1 KO mice, which is reflected in spleen weight and GFP+ cells in the spleen (*N* = 5). Representative flow cytometry demonstrates reduced leukemic GFP+ cells in the spleens from Ifngr1 null mice, a reduction in levels of Ly6C+/CD11b + MDSC and an increase in CD4+/CD8+ cells compared with wild type mice engrafted with BBC2 cells (**D**; N = 5). These changes are quantified in (**E**). Western blot analysis (**F**, above) and genomic DNA PCR (**F**, below) identifies IFNG knockout in BBC2 cell clones #25 and #48. Cell cycle analysis shows that IFNG KO cells do not show any differences in cell cycle progression (**G**). Mice xenografted with these two KO clones show extended survival compared with MC cells engfraftment group, which is reflected in spleen weight and WBC counts (**H**; N = 5). Immunophenotyping at the time of sacrifice (**I** and **J**) shows a significant decrease in GFP+ leukemia cells in the KO#25 cell engraftyed mice compared with MC. These same KO cell engrafted mice showed a significant increase in CD4+ and CD8+ cells and a significant reduction in levels of CD11b+/Ly6C^hi^ mMDSCs and CD11b+/Ly6C^int^ gMDSCs. ns = not significant, * *p* < 0.05, ** *p* < 0.01, *** *p* < 0.001, **** *p* < 0.0001

Given that IFN-γ produced by leukemia cells may function directly on the host immune system to mediate immune evasion, we first investigated the expression pattern of its receptor IFNGR1 in different cell types. Protein expression database searches revealed that the IFNGR1 is predominantly expressed [39] in granulocytes and monocytes (Fig. 7A). Further, flow cytometry analysis of cell-specific expression in BALB/c mice shows predominant expression in CD11b + Ly6C^hi^ and CD11b + Ly6C^int^ myeloid cells, compared to the CD4+/CD8+ T-cells (Fig. 7B). To explore the involvement of IFN-γ in suppressing immune surveillance further, we used an Ifngr1 KO mouse generated in the BALB/c background [40]. When syngeneic BBC2 cells were engrafted, survival was significantly longer in the KO mice compared with wild type mice (Fig. 7C). Spleen and liver weight as well as white blood cell (WBC) counts in the Ifngr1 KO mice also demonstrated attenuated disease progression compared with wild type mice (Fig. 6C). Significantly, the KO mice show reduced levels of MDSCs and increased levels of CD4+/CD8+ cells (Fig. 7D and E), which is consistent with observations from mice engrafted with IRAK1 null cells. These observations further suport a role for IRAK1-regulated IFN-γ in facilitating the induction of MDSCs to establish an immune suppressive microenvironment and promote SCLL progression, but which is lost in the IRAK1 KO cells. Our previous ELISA assay (Fig. 6D) showed that IFN-γ produced by the leukemia cells was a major source of serum IFN-γ. To explore the direct role of leukemia cell derived IFN-γ in immune evasion, we used CRISPR/Ca9 to create IFNG KO clones in the BBC2 cells and generated two complete KO clones that are confirmed at both the protein (Fig. 7F, above) and genomic DNA (Fig. 7F, below) levels. Knockout of IFNG did not have any effect on the percentage of cells in the G1/S/G2/M stages of the cell cycle demonstrating the KO did not affect cell proliferation (Fig. 7G). When these cells were engrafted into BALB/c mice, survival was significantly enhanced compared with the mice engrafted with MC BBC2 cels (Fig. 7H), which was supported by spleen weight and WBC counts (Fig. 7H). Immuno-phenotyping of PB cells in these mice showed decreases in GFP+ cells, as well as gMDSC and mMDSC, with increases in the CD4+ and CD8+ populations in the KO#25 cells compared with the MC engrafted mice (Fig. 7I-J), further confirming the role of IRAK1 activated IFN-γ from leukemia cells in induction of MDSCs during the establishment of immune evasion.

### Targeting IRAK1 reverses immune suppression and attenuates SCLL leukemic development

**Fig. 8:**
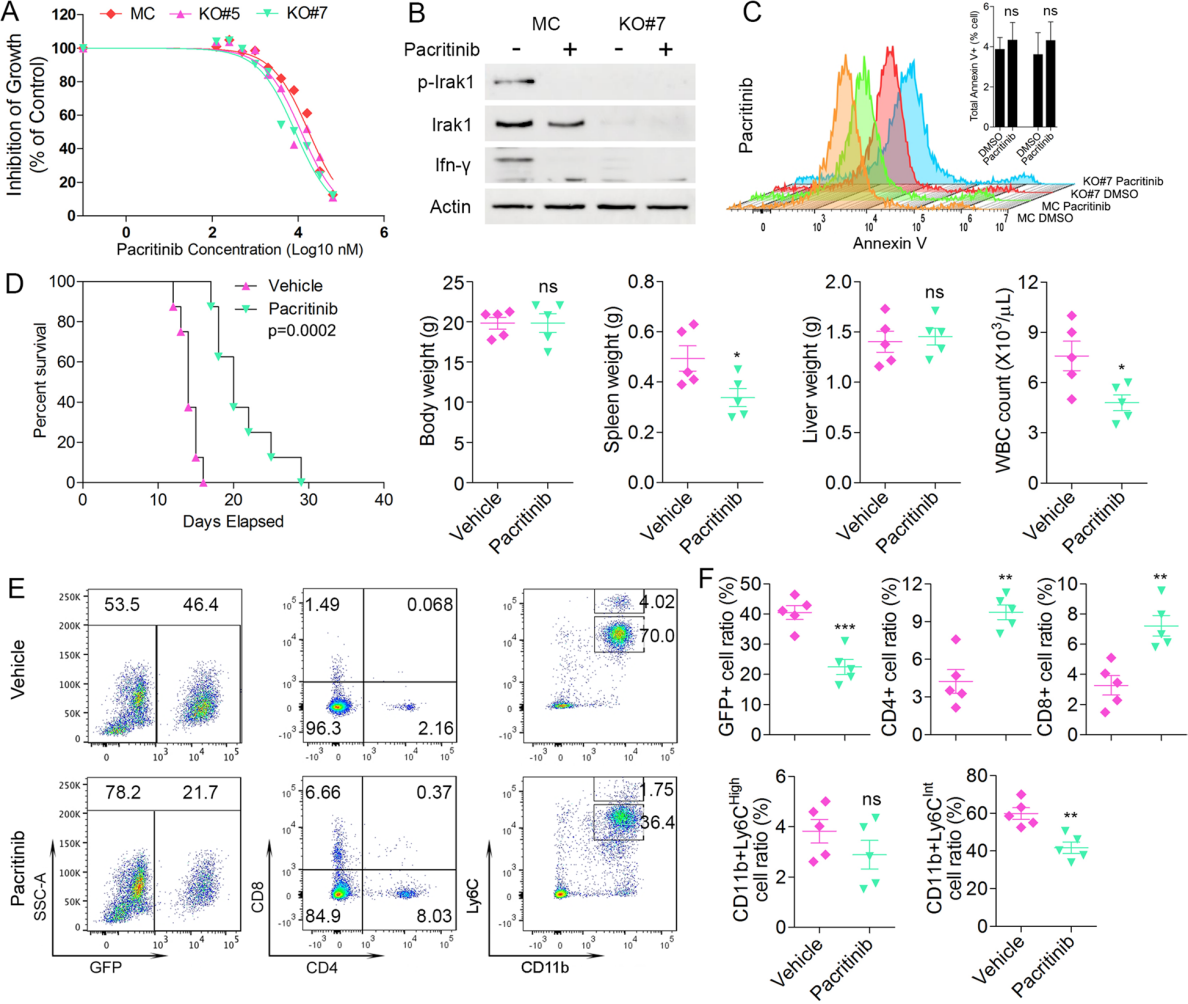
Treatment of MC BBC2 cells and IRAK1 KO #5 and #7 cells (N = 3) with pacritinib shows no differential effects on cell growth inhibition at high doses as determined using CellTiter-Glo Cell Viability Assays (**A**). Pacritinib treatment of the MC cells which express IRAK1 leads to suppression of its phosphactivation and loss of IFN-γ production (**B**). Flow cytometry analysis of annexin V staining (N = 3) of the same cells from (**B**) shows no differential induction of apoptosis in either the MC of KO Cells (**C**). When BBC2 cells were xenografted into BALB/c mice (N = 8) and then treated with the pacritinib IRAK1 inhibitor, there was a significant increase in survival time (**D**), which was reflected in a significantly reduced spleen size and reduction in white blood cell count in the treated cohort. Representative flow cytometry analysis of peripheral blood from the treated and control cells (**E**) at the time of sacrifice shows reduced levels of GFP+ cells in the drug-treated mice (N = 5) and a significantly higher level of CD4+/CD8+ T-cells (**F**). Levels of MDSC in the drug treated mice was also reduced compared with mice treated with vehicle alone. ns = not significant, * *p* < 0.05, ** *p* < 0.01, *** *p* < 0.001

Pacritinib was originally developed as a dual inhibitor of FLT3/JAK2 but was recently shown to also robustly and specifically inhibit IRAK1 [41, 42] and has shown some efficacy in treating AML [33, 43]. When MC cells which express IRAK1 and KO#7 and #9 cells which do not express IRAK1 are treated with pacritinib in vitro there is no differential inhibition of growth (Fig. 8A) even though effective blockage of IRAK1 phospsphoactivation and IFN-γ production is observed at a dose of 1 μM (Fig. 8B). Similarly, there is no differential induction of apoptosis as a result of pacritinib treatment in MC and KO cells (Fig. 8C). These data further confirmed that this IRAK1 signaling does not contribute to leukemia progression through regulation of cell viability and proliferation. In the BBC2 xenograft model of SCLL, however, pacritinib treatment showed a significant (*p* = 0.0002) improvement in survival (Fig. 8D). An ~ 50% reduction in the GFP+ leukemic burden was seen in the pacritinib treated mice as well as a significant reduction in spleen weight and WBC counts (Fig. 8D). In addition, pacritinib treated mice also showed a significant decrease in MDSC levels in the peripheral blood with a concomitant increase in CD4+/CD8+ cells (Fig. 8E-F). These results are consistent with the observations in vivo, where KO of IRAK1 in BBC2 and ZNF112 cells led to decreased MDSCs and increased CD4+/CD8+ T cell levels when xenografted into syngeneic mice. Thus, we demonstrate that, since suppressing IRAK1 expression has no effect on intrinsic cell survival in the cancer cells, its main effect is on modulating immune surveillance in the host, indicating that pharmacological inhibition of IRAK1 may be a promising way to restore immune surveillance against SCLL.

## Discussion

Constitutive activation of FGFR1 kinase promotes transformation of hematopoietic stem cells to initiate SCLL as a result of extensive modulation of gene expression [4–6] and miRNA profiles [7]. While activating miRNAs normally suppresses the function of their target genes, the consequence of FGFR1 activation on Dnmt1 expression [9] broadens its effect by influencing gene suppression through methylation of target sites. We now demonstrate that FGFR1-driven methylation mediates suppression of miR-146b-5p, which leads to tumor development as a result of increased IRAK1 expression. IRAK1 has a well-established role in innate immunity and inflammation and has been implicated in many types of neoplasia, as well as other diseases [11, 12].

The miR146 family comprises two members, miR146a and miR146b, which occupy distinct regions of the genome [10]. The -5p strand in both cases represents the bio-productive strand and they differ in the mature strand by only two nucleotides [44]. Despite this high homology, each performs distinct functions, due to regulation by different transcription factors/co-factors and also distinct tissue expression profiles. Both family members have been implicated in inflammation in various ways, including regulation of toll-like receptor signaling and the innate immune response and both have been shown to regulate IRAK1 and TRAF6. In retinal epithelial cells, however, upregulation of miR146a was dependent on interleukin-1b whereas miR146b was maximally upregulated by interferon gamma [45], suggesting possible unique roles in regulation of immune responses. In this study, it appears that only miR146b is affected by loss of FGFR1 activation, suggesting a further difference between the regulations of the two family members.

In leukemias, deregulated IRAK signaling has been reported in activated B-cell-like diffuse large B-cell lymphoma (ABC DLBCL), myelodysplastic syndrome (MDS) and acute myeloid leukemia (AML) [33, 46, 47]. IRAK1 mRNA is overexpressed in ~ 20–30% of MDS patients, with protein overexpressed and hyperactivated in MDS marrow samples. Most previous investigations implicate IRAK1 in tumor cell survival through its anti-apoptosis effect, as pharmacological inhibition or RNAi knockdown of IRAK1 results in apoptosis [33, 46]. In both melanoma and T cell acute lymphoblastic leukemia (T-ALL), a small-molecule IRAK1/4 inhibitor suppressed cell proliferation and enhanced chemotherapeutic responses [48, 49]. In contrast, IRAK1 knock out in SCLL cells had no significant effect on cell proliferation or apoptosis in vitro but had a profound restrictive effect on leukemia development in vivo. From our studies it is clear that induction of MDSCs and, in turn, suppression of T-cells underlies one of the mechanisms of leukemogenesis driven by FGFR1 kinase.

Pacritinib was developed as an inhibitor of JAK2 and FLT3, which are genes significantly implicated in leukemogenesis and is effective at nanomolar concentrations with high affinity binding to the kinase domain [22, 43]. Although several IRAK1 inhibitors have been developed, only pacritinib is in advanced stage clinical trials [11, 43]. Primary AML cells were sensitive to IRAK inhibition but not other JAK/FLT3 inhibitors, supporting the importance of IRAK1 in these cells [43]. Kinase domain mutations in IRAK1 led to pacritinib resistance, further supporting its importance in AML development. In SCLL, therefore, pacritinib provides another potential therapeutic agent to complement others suggested over the past few years [50]. In addition, the previously proposed mechanism of IRAK1 inhibitors has focussed on cell proliferation and viability, whereas pacritinib treatment in our study demonstrates a novel functional role of IRAK1-mediated inflammatory signaling in the recruitment of MDSCs. Of note, however, there are differences in the survival of mice beween engraftment with IRAK1 KO cells and inhibitor treatment of MC BBC2 cells, which is related to technical limitations. The IRAK1 KO in the leukemia cells is very specific and highly efficient in depleting the target gene specifically in the leukemia cells, while IRAK1 inhibitor treatment has relatively low efficiency, due to the method of delivery. In addition, IRAK1 inhibitors may also block the IRAK1 signaling in the host immune cells including CD4+/CD8+ T cells, and affect their activation and antitumor immune response.

Circulating MDSC levels increase in cancer patients and have a direct effect on lowering survival and suppression of T-cells which, in a cancer setting, prevents tumor clearance. It appears from our fairly detailed survey of cytokines and chemokines that IFN-γ is one of the effectors for tumor cell immune evasion in SCLL, which is regulated by IRAK1 signaling. IFN-γ has traditionally been associated with suppressing tumorigenesis, through enhancing the activity of cells involved in antitumor responses such as T-cells, NK cells, macrophages and dendritic cells [51]. It also stimulates expression of MHC proteins that can present neoantigens from the tumor cells or can act directly on tumor cells by suppressing proliferation and promoting apoptosis. This antitumor activity of IFN-γ is being challenged by emerging facts that no significant improvement for patients was observed in clinical trials of IFN-γ treatment for melanoma and a wide variety of solid tumors as well as leukemia [51], suggesting a dual role that may also favor tumor immune evasion. There is significant evidence that tumor cells can take advantage of IFN-γ as an inducer of pro-tumor effects, through upregulation of IDO and PD-L1 expression in cancer, stromal and myeloid cells and recruitment of Tregs cell and MDSCs to avoid immune recognition and impair the antitumor immunity of effector T cell [51]. We now demonstrated in the SCLL model presented here, however, IFN-γ secretion by the leukemic cells promotes tumor development by inducing MDSCs as part of an IRAK1-dependent mechanism. IFN-γ, however, has different roles depending on the tissue involved. It has been reported that IFN-γ in the bone marrow has a direct effect on hematopoiesis, promoting myelopoiesis and suppressing lymphopoiesis [52]. Since the bone marrow is the primary site for leukemia cell engraftment, > 90% of the bone marrow in late-stage leukemic mice are leukemic cells that produce IFN-γ. This overwhelming IFN-γ enrichied environment could inhibit T cell dfifferentiation and lead to the reduction in T-cells in the peripheral blood in these leukemia mice. IFN-γ also has a functional role in the peripheral blood where the increased levels of IFN-γ result in activation and licencing of MDSCs [35] which in turn suppresses activation of T-cells. These combined effects of IFN-γ may account for the observations in our model system of reduced T-cell and increased MDSCs in the peripheral blood as well as suppression of T-cell function by the increased numbers of activated MDSCs. IFN-γ has been reported to promots the activity of CD4 T helper type 1 cells, while inhibiting Treg cells, Th2 and Th17 differentiation and functions [51], which is consistent with our observation here. Taken together, we show that IRAK1-regulated IFN-γ induced accumulation of MDSCs, which suppresses the T-cell response to the leukemic cells. In total, these observations revealed a previously unknown mechanism how fusion kinases, through the regulation of proinflammatory cytokines, can establish an immune suppressive microenvironment to promote leukemia progression, and provides new potential targets for the treatment of SCLL.

## Conclusions

In conclusion we have demonstrated that constitutive activation of FGFR1 in mouse hematopoietic stem leads to upregulation of IRAK1 as a result of suppression of miR146. Deletion of IRAK1 in SCLL cells leads to suppression of leukemogenesis when xenografted into syngeneic host mice as a result of upregulation of MDSC and a consequential downregulation of CD4+/CD8+ T-cells. This effect on immune surveillance is mediated through upregulation of IFN-γ. Thus IRAK1 appears to be able to modulate the host cell immune environment to permit tumor progression.

## Supplementary Information

**Additional file 1: Supplemental Figure 1.** Conserved target sites for miR-146b-5p across species are shown in (A). Over expression of miR-146b-5p in either murine BBC2 cells, or human KG1 cells, compared with cells expressing the empty vector (EV) shows a > 100-fold increases (B).

**Additional file 2: Supplemental Figure 2.** Schematic representation of the target sites used for the CRISPR/ Cas9 deletion of exons 1–12 in the IRAK1 gene (PAM = protospacer adjacent motif) and showing the location (inverse arrows) of primers (FP and RP) to validate successful deletion of the target region within the deleted region (A). Western blot analysis (B) of 18 BBC2 targeted clones identifies three (#5, #7, and #16) showing no IRAK1 protein, which was validated (C) using genomic DNA PCR with the primers shown in (A). Irak1-targeted ZNF112 cell knockout clones #9, #13, #15and #16 were generated with CRISPR/Cas9, as identified by western blotting (D), which was further confirmed using PCR (E). When injected into BALB/c hosts none of the mice receiving KO #9 or #13 developed leukemia, compared with mice inoculated with sgRNA scrambled (MC) constructs (F, left), which is reflected in spleen weight in these animals at sacrifice (F, right). When the same cells were injected into NSG mice, tumor development was seen in all cases (G, left), which was reflected in the spleen weights from the individual cohorts (*N* = 5). * *p* = < 0.01, ** *p* = < 0.001, ns = not significant.

**Additional file 3: Supplemental Figure 3.** Analysis of NK cells in peripheral blood samples using the CD49b + or CD314+ markers shows no differences between mice engrafted with KO#7 cells and naïve control mice (A and B). Similarly, there were no differences in the levels of γ/δ T-cells (TCR γ/δ+/CD3+) or CD4+ and CD8+ cells (B). Intracellular staining for different cytokins shows that, following in vitro activation using the BioLegend cell activator cocktail, there is no significant increase in levels of the Th2 cytokines IL4 or IL3 in CD4+ cells from the spleens compared with untreated naive control mice (C-D). Similarly, there is no difference in the Th17 cytokines IL-17A or IL22 (E-F).

**Additional file 4: Supplemental Figure 4.** Flow cytometric analysis of disease development in the bone marrow cells following engraftment of either MC BBC2 cells or KO clone#7 shows high levels of GFP+ cells in the bone marrow from MC cell engrafted mice but not in in those engrafted with either KO clone #7 (A-B) or naïve wild type mice. There is a significant increase in levels of Ly6C+/CD11b + MDSC in the mice engrafted with MC cells. In contrast to the spleen, there are no significant changes in the levels of CD4+/CD8+ T-cells in the bone marrow. The relative frequencies of these immune cell types in the KO cell engrafted mice is similar to those seen in naïve wild type mice. Analysis of T-regs in the peripheral blood (C and D) shows no differences between CD4 + Foxp3+ cells from the MC engrafted mice compared with either mice engrafted with KO#7 cells or naïve mice. While there was an increase in CD11b + myeloid cells in the MC engrafted mice, the F4/80+ or CD68+ macrophages showed no differences between the three groups of mice (C and D). Analysis of GFP+ cells from the PB of mice injected with MC ZNF112 cells shows high levels of leukemic cells but virtually none are seen in mice injected with the ZNF112 KO#9 cells (C). In the same mice, there is a ~ 100% increase in the proportion of MDSCs and an ~ 60–70% decrease in CD4+/CD8+ T cells in the mice engrafted with MC cells compared with those engrafted with KO #9 cells (D). * p = < 0.01, ** *p* = < 0.001. *** *p* = < 0.0001, **** *p* = < 0.00001. ns = not significant.

**Additional file 5: Supplemental Figure 5.** Western blot analysis of IRAK1 related signaling molecules (A) shows impaired activated of AKT and p38 in Irak1 KO clones #5 and #7 compared with MC BBC2 cells in response to IL-1β stimulation. Summary of the secretion levels of immune mediators analysed in this study with the BioLegend’s LEGENDplex™ bead-based immunoassays using either parental BBC2 cells or KO clones #5 and #7 in response to stimulation by either LPS or IL-1β for 24 h or 48 h (B). The dotted lines in each case indicate the minimum detectable levels for each of these proteins in this assay. Analysis of RNA levels (*N* = 3) for PD-L1 shows expression levels that correspond to levels of IFN-γ expression (C, above), but there is no change or induction of PD-L2. This relationship was confirmed using flow cytometry (C, below). Analysis of PD-1 expression in CD4+ and CD8+ cells derived from mice xenografted with MC BBC2 cells compared with wild type naïve mice (D) shows no significant difference between the two groups.

## Abbreviations

SCLL: Stem Cell Leukemia/Lymphoma syndrome
FGFR1: Fibroblast growth factor receptor 1
CRISPR: Clustered regularly interspaced short palindromic repeats
IRAK1: Interleukin receptor associated kinase
MDSC: Myeloid-derived suppressor cells
IFN: Interferon
SRC: v-src avian sarcoma (Schmidt-Ruppin A-2) viral oncogene homolog
PLCG: Phospholipase C gamma
STAT: Signal transduced and activator of treanscription
AML: Acute myelogenous leukemia
BAX: BCL2-associated protein
BCL2: B-cell CLL/lymphoma 2
MYB: v-myb avian myeloblastosis viral oncogene homolog
Lps: Lipopolysaccharide
Il1r: Interleukin 1 receptor
Tlr: Toll like receptor
TRAF: TNF receptor associated factor
MAPK: Mitogen activated protein kinase
IRNGR1: Interferon gamm receptor 1
PB: Peripheral blood
DNMT: DNA methyltransferase
UTR: Untranslated region
BCR: Breakpoint cluster region
MC: Mock control
NSG: Nod/Scid/interleukin gamma deficient mice
KO: Knock out
GFP: Green fluorescent protein
ALL: Acute lymphocytic leukemia
PD-L1: Programed cell death like 1
FLT: Fms related tyrosine kinase 3
JAK: Janus kinase
MDS: Myelodysplastic syndrome
