# Supplementary figures and images for: IRAK1-regulated IFN-γ signaling induces MDSC to facilitate immune evasion in FGFR1-driven hematological malignancies
Source: Mol Cancer. 2021 Dec 14;20:165. doi: 10.1186/s12943-021-01460-1 (PMC8670266; doi:10.1186/s12943-021-01460-1)

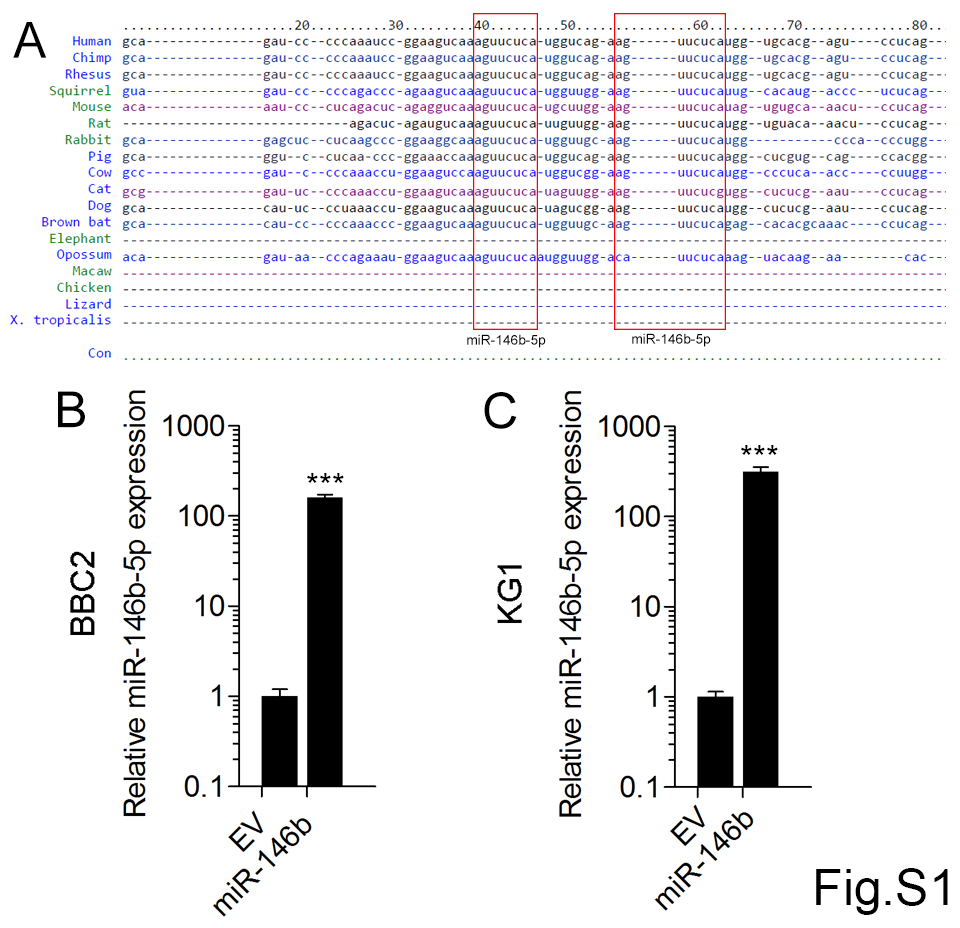

Supplement: Supplementary file 1 — Additional file 1: Supplemental Figure 1. Conserved target sites for miR-146b-5p across species are shown in (A). Over expression of miR-146b-5p in either murine BBC2 cells, or human KG1 cells, compared with cells expressing the empty vector (EV) shows a > 100-fold increases (B). [file 12943_2021_1460_MOESM1_ESM.tif]

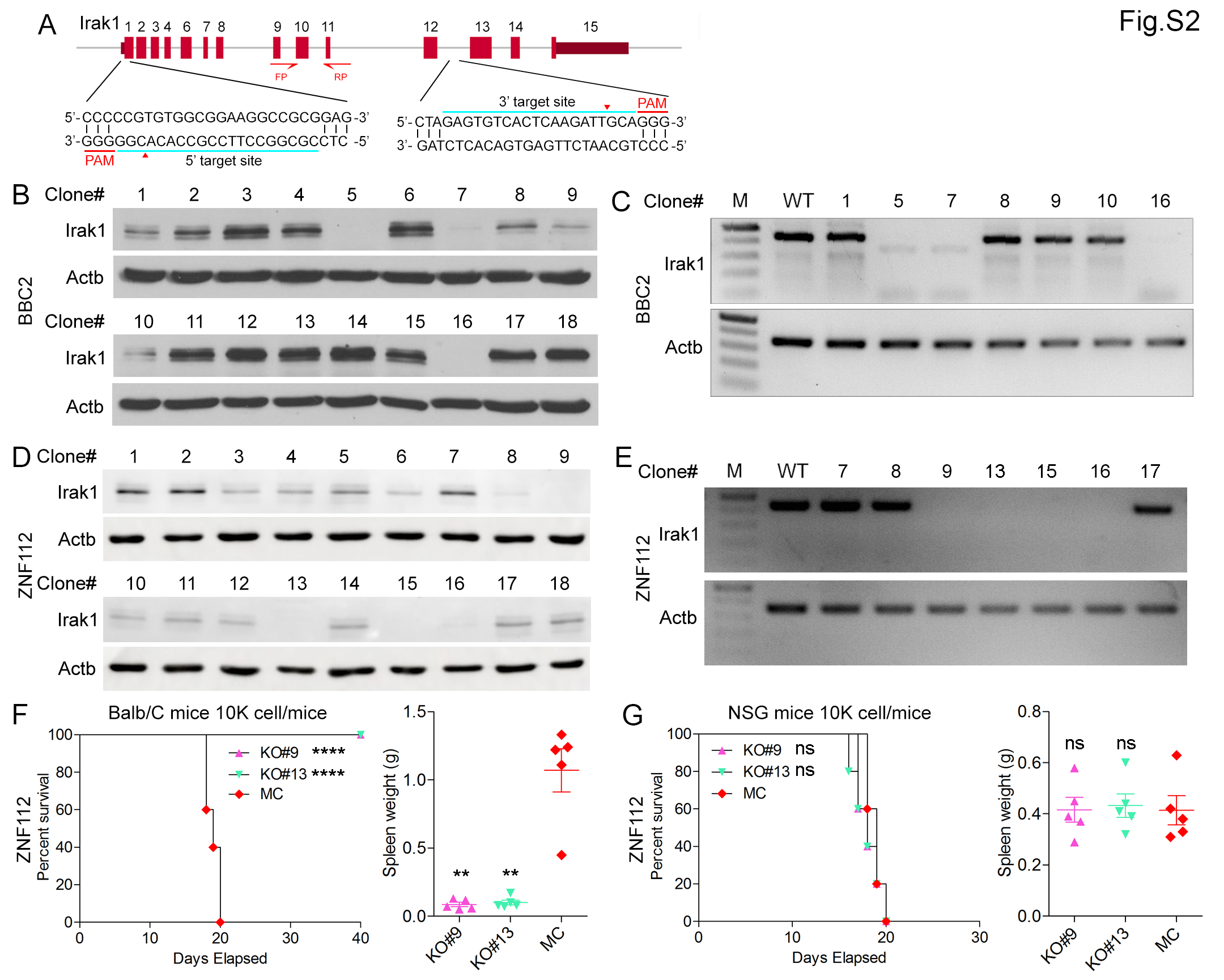

Supplement: Supplementary file 2 — Additional file 2: Supplemental Figure 2. Schematic representation of the target sites used for the CRISPR/ Cas9 deletion of exons 1–12 in the IRAK1 gene (PAM = protospacer adjacent motif) and showing the location (inverse arrows) of primers (FP and RP) to validate successful deletion of the target region within the deleted region (A). Western blot analysis (B) of 18 BBC2 targeted clones identifies three (#5, #7, and #16) showing no IRAK1 protein, which was validated (C) using genomic DNA PCR with the primers shown in (A). Irak1-targeted ZNF112 cell knockout clones #9, #13, #15and #16 were generated with CRISPR/Cas9, as identified by western blotting (D), which was further confirmed using PCR (E). When injected into BALB/c hosts none of the mice receiving KO #9 or #13 developed leukemia, compared with mice inoculated with sgRNA scrambled (MC) constructs (F, left), which is reflected in spleen weight in these animals at sacrifice (F, right). When the same cells were injected into NSG mice, tumor development was seen in all cases (G, left), which was reflected in the spleen weights from the individual cohorts (N = 5). * p = < 0.01, ** p = < 0.001, ns = not significant. [file 12943_2021_1460_MOESM2_ESM.tif]

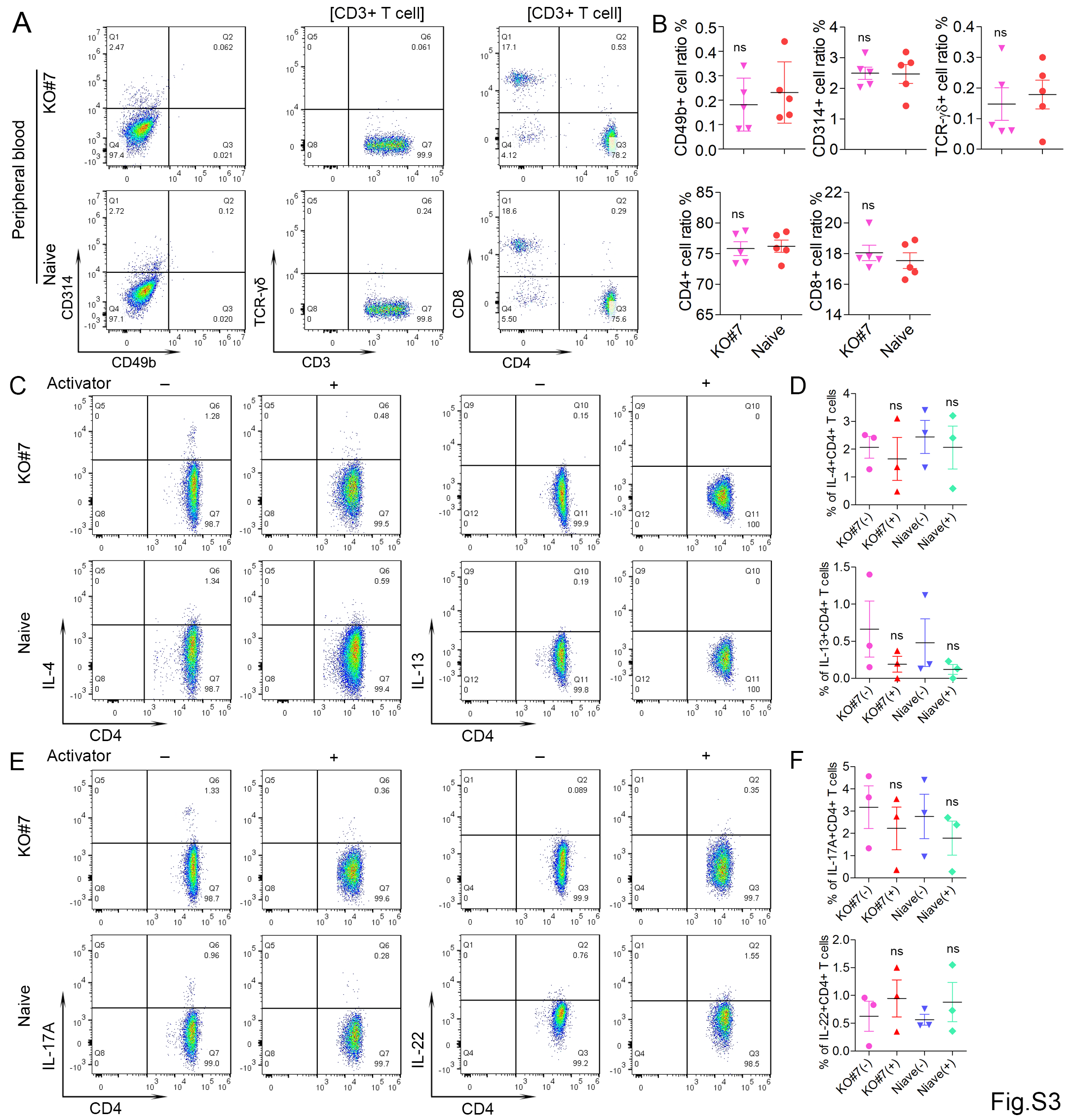

Supplement: Supplementary file 3 — Additional file 3: Supplemental Figure 3. Analysis of NK cells in peripheral blood samples using the CD49b + or CD314+ markers shows no differences between mice engrafted with KO#7 cells and naïve control mice (A and B). Similarly, there were no differences in the levels of γ/δ T-cells (TCR γ/δ+/CD3+) or CD4+ and CD8+ cells (B). Intracellular staining for different cytokins shows that, following in vitro activation using the BioLegend cell activator cocktail, there is no significant increase in levels of the Th2 cytokines IL4 or IL3 in CD4+ cells from the spleens compared with untreated naive control mice (C-D). Similarly, there is no difference in the Th17 cytokines IL-17A or IL22 (E-F). [file 12943_2021_1460_MOESM3_ESM.tif]

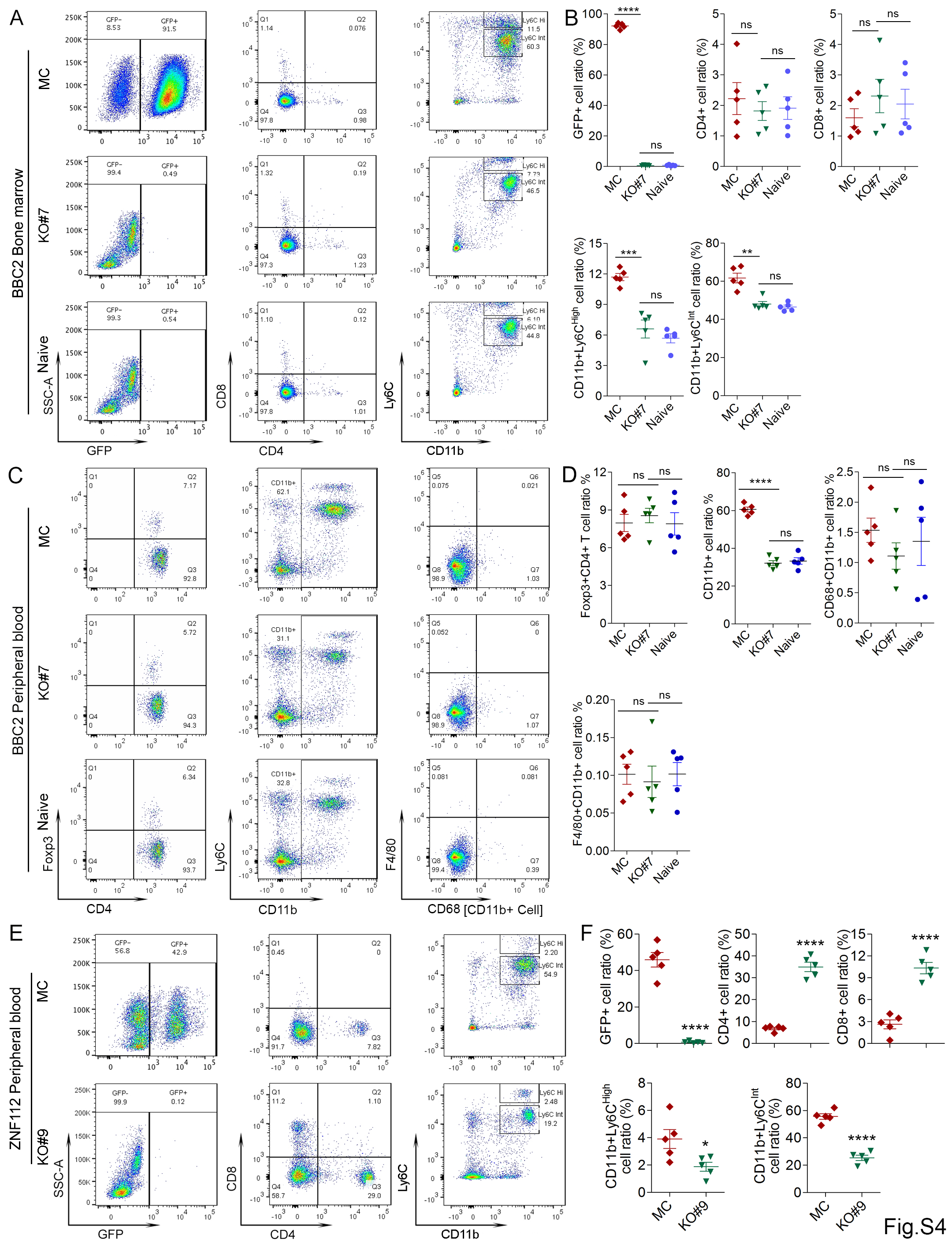

Supplement: Supplementary file 4 — Additional file 4: Supplemental Figure 4. Flow cytometric analysis of disease development in the bone marrow cells following engraftment of either MC BBC2 cells or KO clone#7 shows high levels of GFP+ cells in the bone marrow from MC cell engrafted mice but not in in those engrafted with either KO clone #7 (A-B) or naïve wild type mice. There is a significant increase in levels of Ly6C+/CD11b + MDSC in the mice engrafted with MC cells. In contrast to the spleen, there are no significant changes in the levels of CD4+/CD8+ T-cells in the bone marrow. The relative frequencies of these immune cell types in the KO cell engrafted mice is similar to those seen in naïve wild type mice. Analysis of T-regs in the peripheral blood (C and D) shows no differences between CD4 + Foxp3+ cells from the MC engrafted mice compared with either mice engrafted with KO#7 cells or naïve mice. While there was an increase in CD11b + myeloid cells in the MC engrafted mice, the F4/80+ or CD68+ macrophages showed no differences between the three groups of mice (C and D). Analysis of GFP+ cells from the PB of mice injected with MC ZNF112 cells shows high levels of leukemic cells but virtually none are seen in mice injected with the ZNF112 KO#9 cells (C). In the same mice, there is a ~ 100% increase in the proportion of MDSCs and an ~ 60–70% decrease in CD4+/CD8+ T cells in the mice engrafted with MC cells compared with those engrafted with KO #9 cells (D). * p = < 0.01, ** p = < 0.001. *** p = < 0.0001, **** p = < 0.00001. ns = not significant. [file 12943_2021_1460_MOESM4_ESM.tif]

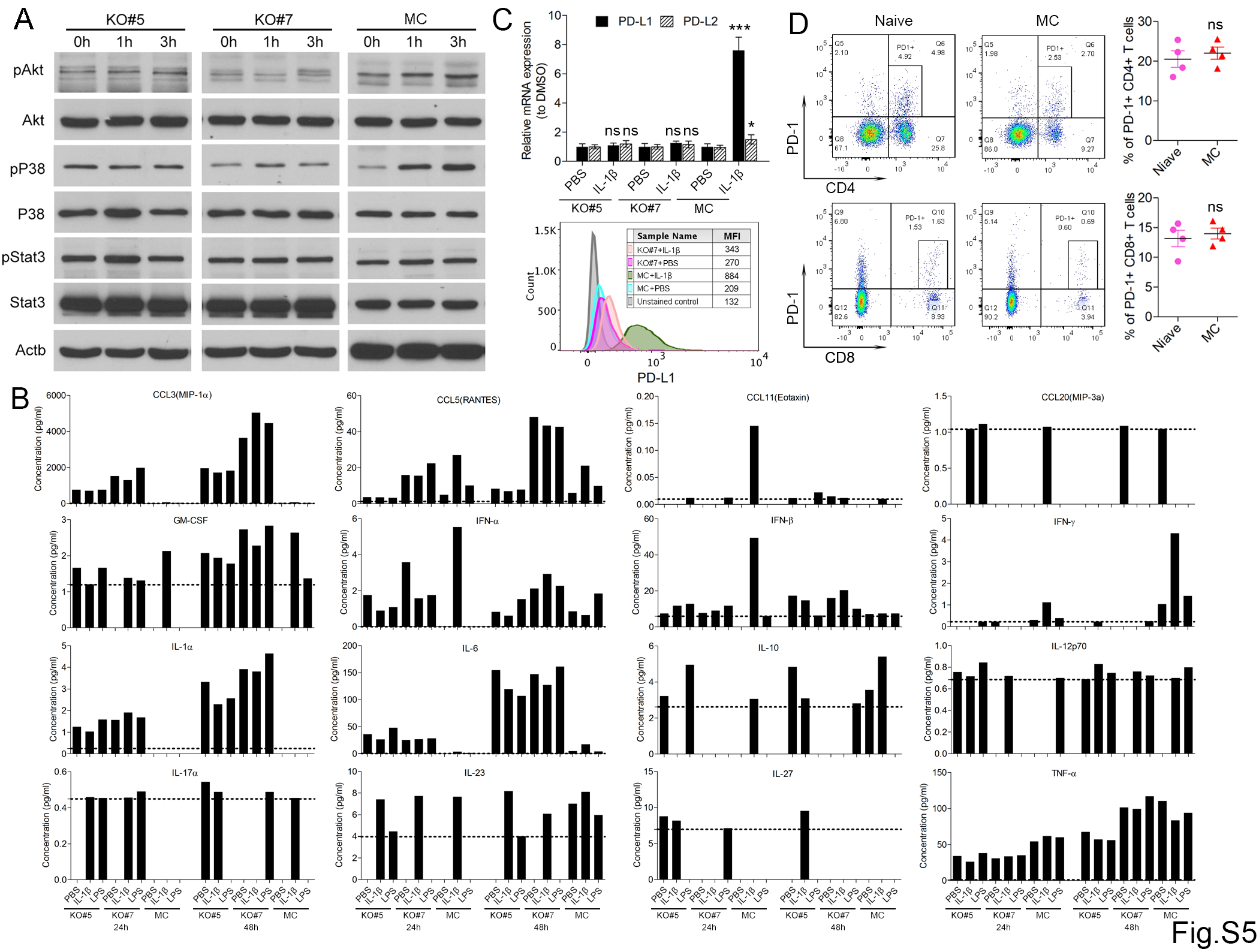

Supplement: Supplementary file 5 — Additional file 5: Supplemental Figure 5. Western blot analysis of IRAK1 related signaling molecules (A) shows impaired activated of AKT and p38 in Irak1 KO clones #5 and #7 compared with MC BBC2 cells in response to IL-1β stimulation. Summary of the secretion levels of immune mediators analysed in this study with the BioLegend’s LEGENDplex™ bead-based immunoassays using either parental BBC2 cells or KO clones #5 and #7 in response to stimulation by either LPS or IL-1β for 24 h or 48 h (B). The dotted lines in each case indicate the minimum detectable levels for each of these proteins in this assay. Analysis of RNA levels (N = 3) for PD-L1 shows expression levels that correspond to levels of IFN-γ expression (C, above), but there is no change or induction of PD-L2. This relationship was confirmed using flow cytometry (C, below). Analysis of PD-1 expression in CD4+ and CD8+ cells derived from mice xenografted with MC BBC2 cells compared with wild type naïve mice (D) shows no significant difference between the two groups. [file 12943_2021_1460_MOESM5_ESM.tif]
